# IQGAP1 promotes pancreatic cancer progression and epithelial-mesenchymal transition (EMT) through Wnt/β-catenin signaling

**DOI:** 10.1038/s41598-019-44048-y

**Published:** 2019-05-17

**Authors:** Wei Hu, Zhongxia Wang, Shan Zhang, Xian Lu, Junyi Wu, Kuanyong Yu, Anlai Ji, Wei Lu, Zhong Wang, Junhua Wu, Chunping Jiang

**Affiliations:** 1 Department of Hepatobiliary Surgery, Lianyungang Clinical College of Nanjing Medical University, Lianyungang, 222001 Jiangsu China; 2 Department of Hepatobiliary Surgery, Drum Tower Clinical College of Nanjing Medical University, Nanjing, 210008 Jiangsu China; 3 Jiangsu Key Laboratory of Molecular Medicine, Medical School, Nanjing University, Nanjing, 210093 Jiangsu China; 4 Jiangsu Province Hospital of Traditional Chinese Medicine, Nanjing, 210029 Jiangsu China

**Keywords:** Pancreatic cancer, RNAi

## Abstract

IQ motif-containing GTPase-activating protein 1 (IQGAP1) is a scaffold protein that participates in several cellular functions, including cytoskeletal regulation, cell adhesion, gene transcription and cell polarization. IQGAP1 has been implicated in the tumorigenesis and progression of several human cancers. However, the role of IQGAP1 in pancreatic ductal adenocarcinoma (PDAC) is still unknown. We found that IQGAP1 expression was an independent prognostic factor for PDAC. IQGAP1 upregulation significantly promoted cell proliferation, migration, invasion and epithelial-mesenchymal transition (EMT), whereas IQGAP1 downregulation impaired its oncogenic functions. Overexpression of IQGAP1 increased the protein level of Dishevelled2 (DVL2) and enhanced canonical Wnt signaling as evidenced by increased DVL2 level, β-catenin transcriptional activity, β-catenin nuclear translocation and expression of the direct target genes of β-catenin (cyclin D1 and c-myc). In contrast, knockdown of IQGAP1 decreased the level of DVL2 and attenuated Wnt/β-catenin signaling. *In vivo* results revealed that IQGAP1 promoted tumor growth and metastasis. Co-immunoprecipitation studies demonstrated that IQGAP1 interacted with both DVL2 and β-catenin. Moreover, knockdown of DVL2 reversed IQGAP1-induced EMT. Our findings thus confirmed that IQGAP1 could be used as a potential target for PDAC treatment.

## Introduction

Pancreatic cancer (PC) remains one of the most lethal malignancies with a 5-year survival rate of less than 5%^1^. Despite progress in treatment strategies, the decrease in mortality rate for this disease is still slow^2^ due to aggressive growth, distant metastasis, chemoresistance and difficulties in early diagnosis^3–5^. Pancreatic ductal adenocarcinoma (PDAC) accounts for more than 95% of all PC cases^6^. Therefore, there is an urgent need to explore diagnostic molecular biomarkers and targets with high therapeutic efficacy to improve the prognosis of PDAC patients.

As one of the evolutionally conserved scaffold protein family members, IQ motif-containing GTPase-activating protein 1 (IQGAP1) includes five domains, namely, the calponin homology domain interacting with F-actin, GAP-related domain interacting with small GTPases (Rac1 and Cdc42), poly-proline protein-protein domain interacting with ERK, IQ domain interacting with four IQ motifs and RasGAP C-terminus^7^. Several cellular functions are regulated by the multidomain structures of IQGAP1 through its binding with many other partners, which is mainly mediated by the RGCT or IQ regions^8,9^. For example, IQGAP1 functions as a scaffold in the MEK/ERK cascade by binding to MEK1/2, ERK1/2 and B-Raf via its IQ region^10^. Accumulating evidence indicates that IQGAP1 is an oncogene^11,12^. IQGAP1 expression has been reported to be upregulated in several types of human cancer tissues and cell lines, such as hepatocellular carcinoma^12^, thyroid cancer^13^, breast cancer^14^, esophageal squamous cell carcinoma^15^, non-small cell lung cancer^16^ and PC^17^.

However, the function and underlying mechanism of IQGAP1 in PDAC have not been fully evaluated. The present study aimed to investigate the prognostic values and biological functions of IQGAP1 and the associated molecular mechanisms in PDAC. We first evaluated the expression level of IQGAP1 in PDAC tissues using immunohistochemistry (IHC) and investigated the association of IQGAP1 expression level with clinicopathological features and patient survival. Then, we studied the role of IQGAP1 in the progression of PDAC by using both *in vivo* and *in vitro* models. Finally, we explored the effect of IQGAP1 on the Wnt/β-catenin signaling pathway in PDAC cells.

## Results

### Expression of IQGAP1 in PDAC tissues is significantly higher than that in adjacent normal tissues

**Figure 1:**
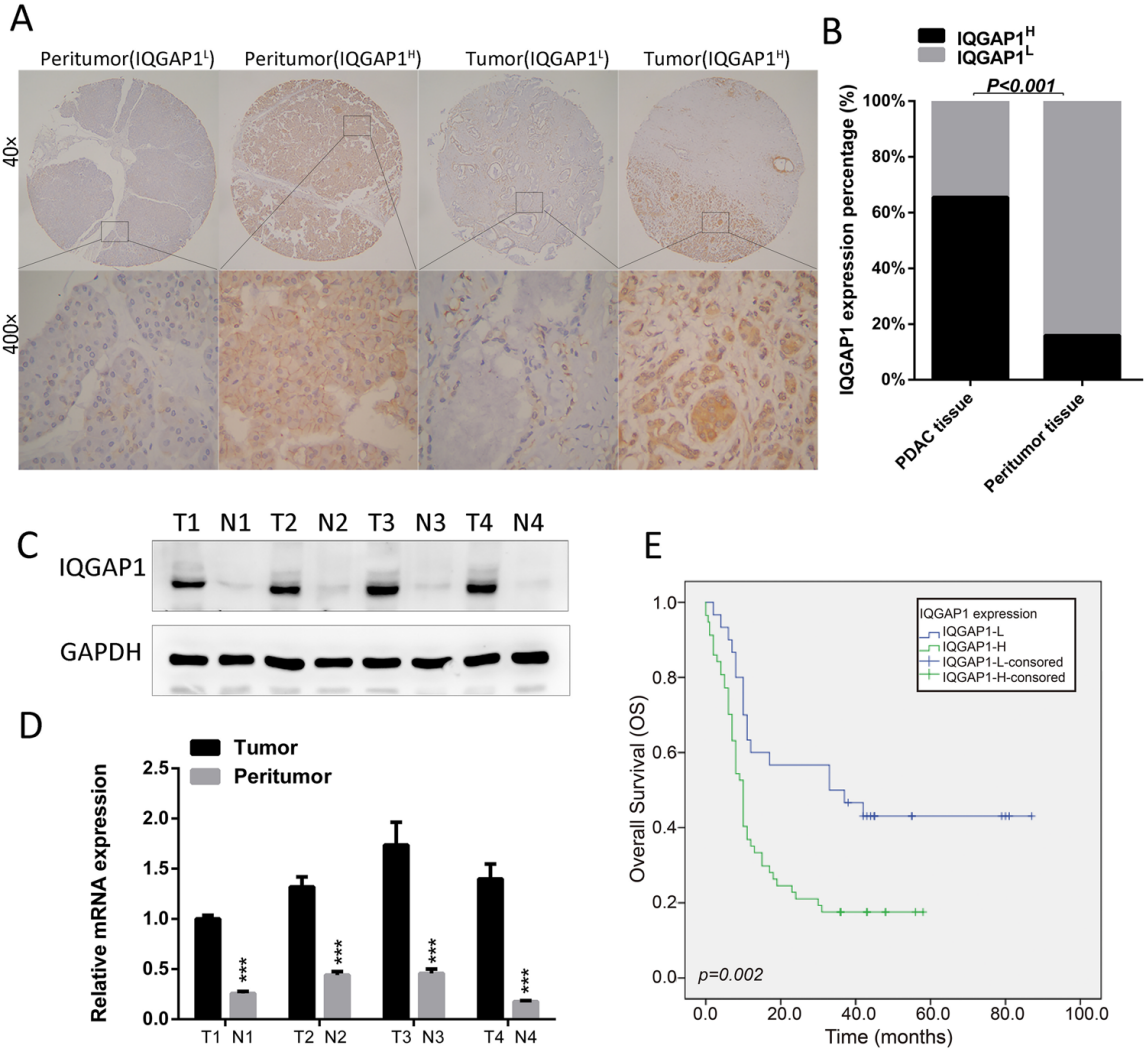
Expression of IQGAP1 in PDAC tissues. (**A**,**B**) Expression of IQGAP1 in PDAC and peritumor tissues in TMAs was detected by IHC. Percentage of high expression of IQGAP1 (SI > 4) was detected in 65.5% and 15.9% of in PDAC tissues and matched peritumor tissues, respectively. (**C**,**D**) Protein and mRNA expression of IQGAP1 was detected in PDAC and paired adjacent tissue samples from 4 different patients. (**E**) High expression of IQGAP1 (SI > 4) was correlated with poor prognosis in PDAC patients. *P < 0.05, **P < 0.01, and ***P < 0.001.

The expression of IQGAP1 in 87 PDAC tissues identified by IHC was classified into two groups: the low-IQGAP1 group (IQGAP1^L^) (that is, staining index (SI) ≤4) containing 30 samples and the high-IQGAP1 group (IQGAP1^H^) (that is, SI >4) containing 57 samples. The percentage of high expression of IQGAP1 (65.5%, 57/87) in PDAC tissues was significantly higher than that (15.9%, 11/ 69) in matched peritumor tissues (P < 0.001, Fig. 1A,B). Compared with that in paired adjacent tissues, the protein and mRNA expression of IQGAP1 in PDAC tissue samples from 4 different patients was upregulated (Fig. 1C,D). The analysis of the relationship between the expression levels of IQGAP1 and clinicopathologic variables showed that IQGAP1 protein expression is significantly correlated with histological differentiation (P = 0.034) and pathological N stage (P = 0.030) (Table 1).

**Table 1:** Correlation between IQGAP1 expression and clinicopathological variables in patients with PDAC.

Variables	Number	IQGAP1^L^ (%)	IQGAP1^H^ (%)	P*
**Sex**				
Male	54	20 (37.0)	34 (63.0)	0.521
Female	33	10 (30.3)	23 (69.7)	
**Age** ^**a**^				
≤61	44	16 (36.4)	28 (63.6)	0.709
>61	43	14 (32.6)	29 (67.4)	
**Tumor location**				
Pancreatic head	56	16 (28.6)	40 (71.4)	0.119
Non-head	31	14 (45.2)	17 (54.8)	
**Tumor size (cm)**				
<4	26	5 (19.2)	21 (80.8)	0.051
≥4	61	25 (41.0)	36 (59.0)	
**Histological differentiation**				
Well-moderate	66	27 (40.9)	39 (59.1)	0.034^b^
Poor	21	3 (14.3)	18 (85.7)	
**TNM stage**				
I + II	86	30 (34.9)	56 (65.1)	1^b^
III + IV	1	0 (0)	1 (100)	
T classification				0.397
T1/T2	68	25 (36.8)	43 (63.2)	
T3	19	5 (26.3)	14 (73.7)	
**N classification**				
N0	47	21 (44.7)	26 (55.3)	0.030
N1	40	9 (22.5)	31 (77.5)	
**M classification**				
M0	86	30 (34.9)	56 (65.1)	1^b^
M1	1	0 (0)	1 (100)	
**Perineural invasion**				
Present	35	10 (28.6)	25 (71.4)	0.341
Absent	52	20 (38.5)	32 (61.5)	

^a^Median age; ^b^Fisher’s exact test.

*P < 0.05 indicates a significant association among the variables.

### IQGAP1 expression is an independent prognostic predictor for PDAC patients

We further investigated whether IQGAP1 can be used as a prognostic indicator in PDAC patients. Only 23 patients were still alive by the last follow-up. Kaplan-Meier analysis and log-rank test were used to explore the relationship between IQGAP1 expression levels and clinical follow-up information in 87 PDAC patients. PDAC patients in the IQGAP1^H^ group had significantly poorer overall survival (OS) rates than patients in the IQGAP1^L^ group (P = 0.002, Fig. 1E). Univariate and multivariate Cox analyses were used to assess the correlations between the immunohistochemical data for IQGAP1 expression and clinicopathologic features (Supplementary Table S1). The median OS of patients in the IQGAP1^H^ group and IQGAP1^L^group was 10.0 months (95% confidence interval [CI] 8.325–11.675) and 33.0 months (95% CI 0.085–65.915), respectively. Univariate analysis indicated that the following variables were significantly related to OS: histological differentiation (P = 0.047), N stage (P < 0.001), and IQGAP1 expression (P = 0.004). In multivariate analysis using Cox proportional hazards models, N1 stage (HR 2.786; 95% CI 1.617–4.800; P < 0.001), poor tumor differentiation (HR 1.969; 95% CI 1.107–3.500; P = 0.021), and high IQGAP1 expression (hazard ratio [HR] 1.880; 95% CI 1.049–3.367; P = 0.034) were correlated with OS (Supplementary Table S1). Therefore, IQGAP1 expression is an independent prognostic predictor in PDAC patients.

### Successful inhibition of IQGAP1 following siRNA transfection in CFPAC-1 and SW1990 cells

**Figure 2:**
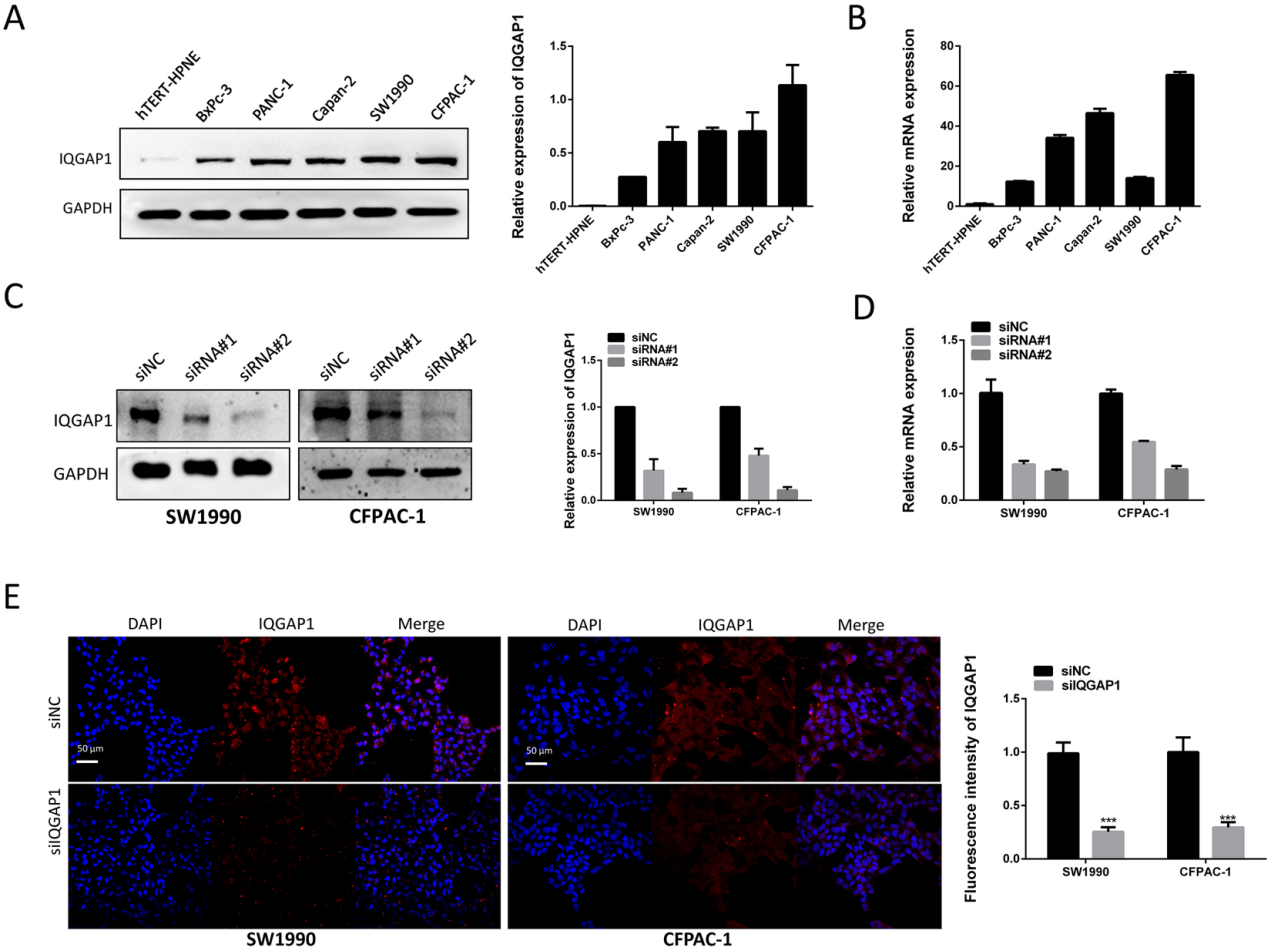
IQGAP1 expression in PC cell lines and interference efficiency of two siRNAs for IQGAP1. (**A**,**B**) Protein and mRNA expression of IQGAP1 was observed in different PC cell lines. (**C**,**D**) Interference efficiency of two siRNAs for IQGAP1 was examined by qRT-PCR and western blotting. (**E**) Immunofluorescence assays showing IQGAP1 expression in siNC- and siIQGAP1-transfected PC cells. IQGAP1 protein is stained in red, and nuclei are stained with DAPI in blue. Uncropped western blots are shown in Supplementary Fig. 2.

The expression of IQGAP1 was detected in BxPc-3, SW1990, Capan-2, CFPAC-1, PANC-1, and hTERT-HPNE cells by qRT-PCR and western blotting. Higher expression of IQGAP1 mRNA and protein was observed in PC cells than in hTERT-HPNE cells and the expression level of IQGAP1 protein in SW1990 and CFPAC-1 cells is the highest among all PC cells (Fig. 2A,B). To further assess the role of IQGAP1 in PDAC, we knocked down IQGAP1 in PC cells. SW1990 and CFPAC-1 cells were selected for IQGAP1 knockdown by siRNA. The interference efficiency of two siRNAs for IQGAP1 was confirmed through comparison with negative controls at both mRNA and protein levels. SiRNA#2 (siIQGAP1) could significantly decrease IQGAP1 mRNA and protein levels and was used for further function and mechanism investigation (Fig. 2C,D). As indicated by immunofluorescence, compared with that in control cells, IQGAP1 expression was downregulated in siRNA#2-transfected cells (Fig. 2E). Secondary antibody only staining was used as negative control (Supplementary Fig. 1A).

### Knockdown of IQGAP1 represses the proliferation, migration and invasion of PC cells

**Figure 3:**
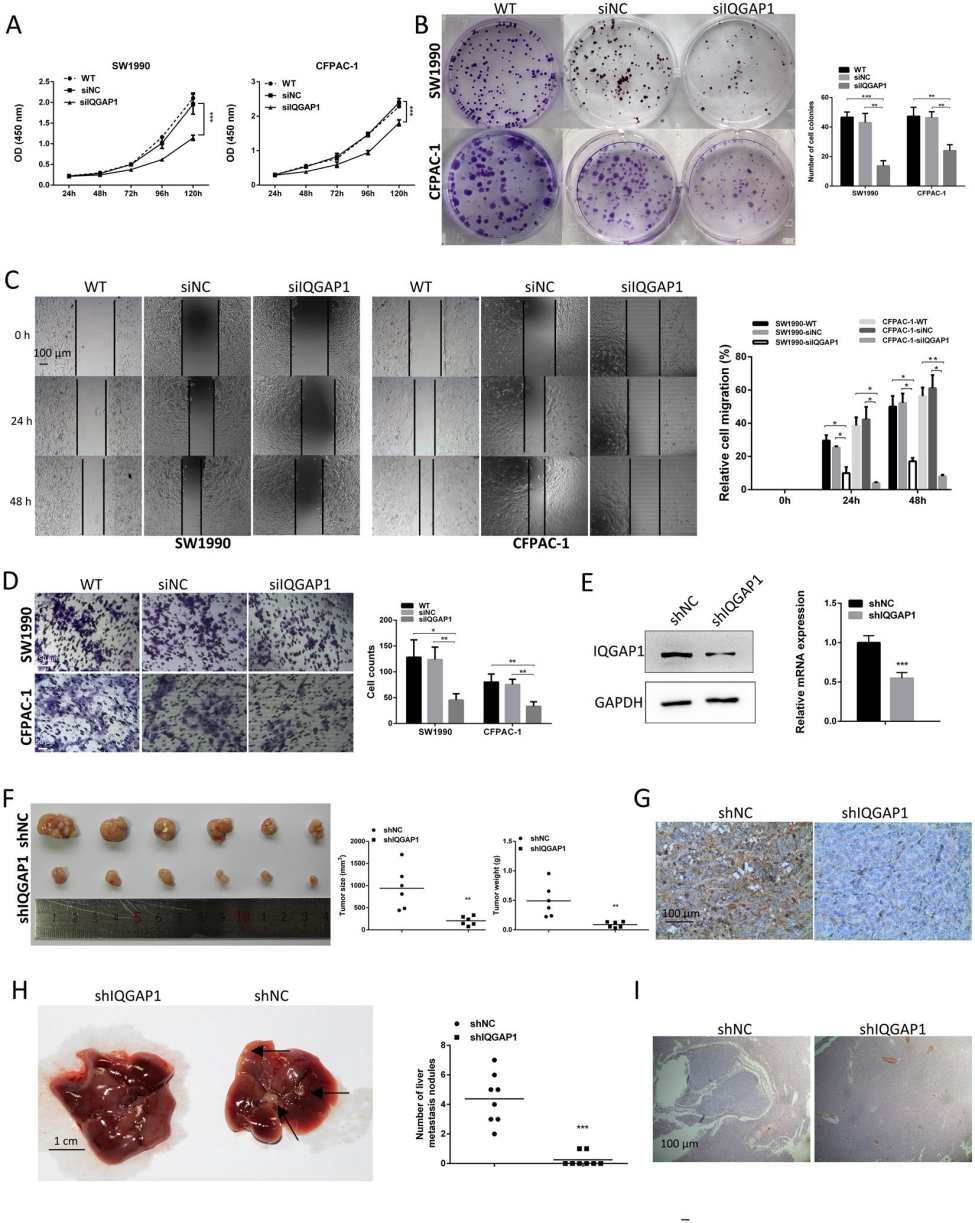
Knockdown of IQGAP1 inhibits the proliferation, migration and invasion of PC cells. Knockdown of IQGAP1 inhibits cell growth, as determined by CCK-8 (**A**) and colony formation assays (**B**). (**C**) Representative images of wound healing by IQGAP1 knockdown (siRNA), control (siNC) and wild-type (WT) cells were photographed at 0, 24 and 48 hours after scraping. (**D**) Cell migration was measured by transwell assays. Number of migrated cells was exhibited as the mean ± SD of three randomly chosen visual fields from three independent experiments. (**E**) Western blotting and qRT-PCR were used to confirm the stable interference of IQGAP1 expression. (**F**) Gross observation of xenograft tumor size. Volume and weight of tumors derived from nude mouse models following IQGAP1 downregulation by shRNA were decreased *in vivo*. (**G**) Xenograft tumor tissues were stained with anti-IQGAP1 antibody by IHC. (**H**) Liver metastatic nodules (black arrow) were evaluated approximately 3 months postinjection (n = 8 per group). (**I**) Representative results of H&E staining of metastatic nodules in the liver. *P < 0.05, **P < 0.01, and ***P < 0.001. Uncropped western blots are shown in Supplementary Fig. 2.

**Figure 4:**
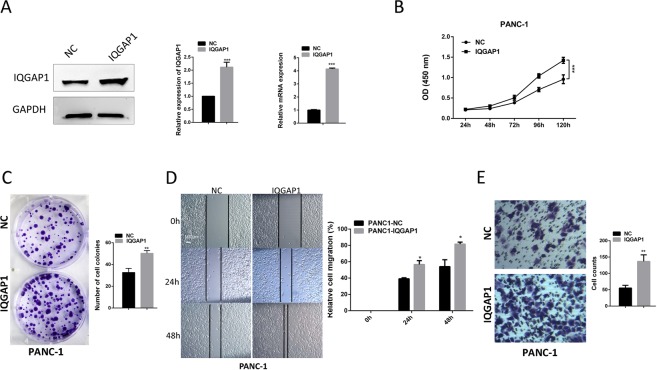
IQGAP1 overexpression promotes the proliferation of PC cells *in vitro*. (**A**) Significant increase in IQGAP1 expression in PANC-1-IQGAP1 cells compared with that in control cells shown by western blotting and qRT-PCR analysis. (**B**,**C**) Viability and colony formation efficiency were significantly higher in PANC-1 cells transfected with IQGAP1 plasmid than in control cells. (**D**) Representative images of wound healing by PANC-1-IQGAP1 and control cells were photographed at 0, 24 and 48 hours after scraping. (**E**) Cell migration was measured by transwell assays. Number of migrated cells was expressed as the mean ± SD of three randomly chosen visual fields from three independent experiments. *P < 0.05, **P < 0.01, and ***P < 0.001. Uncropped western blots are shown in Supplementary Fig. 2.

Given that high IQGAP1 expression was significantly related to lymph node metastasis in PC tissues (Table 1), we hypothesized that IQGAP1 may promote the proliferation, migration and invasion of PC cells. Indeed, knockdown of IQGAP1 markedly inhibited cell growth and cell proliferation (Fig. 3A,B). Both wound healing assay and transwell assay are used to observe the migration ability. To prevent potential confounding factors brought by cell proliferation, we examined in detail the difference of proliferation between cells with different transfection conditions. Firstly, raw optical density data from CCK-8 assay indicated that at 24 h (the time point when cells were subjected to wound healing assay), no significant difference of cell proliferation was observed between cells with different IQGAP1 expression conditions (Figs 3A, 4B). Secondly, during wound healing assay, cells were cultured under serum-free condition, which further eliminated potential effect of cell proliferation on migration. Lastly, proliferation assay mimicking culture condition during wound healing assay showed little difference of cell proliferation (Supplementary Fig. 1B). Our results showed that cell migration was markedly inhibited in IQGAP1 knockdown cells as compared with control cells (Fig. 3C,D). The effect of IQGAP1 silencing on migratory ability in CFPAC-1 cells seemed more pronounced than in SW1990 in wound healing assays, while the opposite occured in transwell migration assays, which maybe resulted from that the size of SW1990 cells is smaller than that of CFPAC-1 cells, so in transwell assays SW1990 cells may pass through the 8-µm chamber easier than CFPAC-1 cells. Western blotting and qRT-PCR were used to confirm the stable interference of IQGAP1 expression (Fig. 3E). *In vivo* xenograft assays confirmed that on day 24, the volume and weight of tumors generated from SW1990 cells with silenced IQGAP1 expression (SW1990-shIQGAP1) were smaller than those of tumors generated from negative control cells (SW1990-shNC) (Fig. 3F) (mean volumes of 205 mm3 versus 942 mm3, respectively). IHC staining showed that pancreatic tumors formed by SW1990-shIQGAP1 cells had lower IQGAP1 expression than those formed by SW1990-shNC cells (Fig. 3G). Additionally, liver metastasis models were constructed by orthotopically injecting shIQGAP1 and shNC cells into the major pancreatic duct of nude mice. Three months after the injection, the mice were euthanized, and liver metastases were counted and fixed in 10% formalin. The average number of visible liver metastatic nodules in the shIQGAP1 group was markedly lower than that in the shNC group (Fig. 3H,I). Therefore, the inhibition of IQGAP1 expression suppresses the proliferation of PC cells *in vitro* and *in vivo*, migration and invasiveness of PC cells *in vitro*, and the metastasis of PC cells *in vivo*.

### Overexpression of IQGAP1 promotes the proliferation and migration of human PC cells

To further assess the oncogenic role of IQGAP1, we next overexpressed IQGAP1 in the PC cell line PANC-1 (PANC-1-IQGAP1 cells) by transiently transfecting PANC-1 cells with IQGAP1 plasmids. Western blotting and qRT-PCR analysis confirmed a significant increase in IQGAP1 expression in PANC-1-IQGAP1 cells relative to that in control cells (Fig. 4A). Overexpression of IQGAP1 in PANC-1 cells induced faster cell growth and stronger migratory capability than those in control cells (Fig. 4B,C). Furthermore, CCK-8 and colony formation assays showed that the PANC-1-IQGAP1 cells demonstrated a considerable growth advantage relative to the respective control cells (Fig. 4B,C). Meanwhile, more accelerated wound healing ability and distinctly higher migration rates were observed in PANC-1-IQGAP1 cells than in the control cells by wound healing assay and transwell assay, respectively (Fig. 4D,E). Thus, IQGAP1 overexpression positively regulates the proliferation and migration of PC cells *in vitro*.

### IQGAP1 interacts with Dishevelled 2 (DVL2) and β-catenin and translocates DVL2 into the nucleus

**Figure 5:**
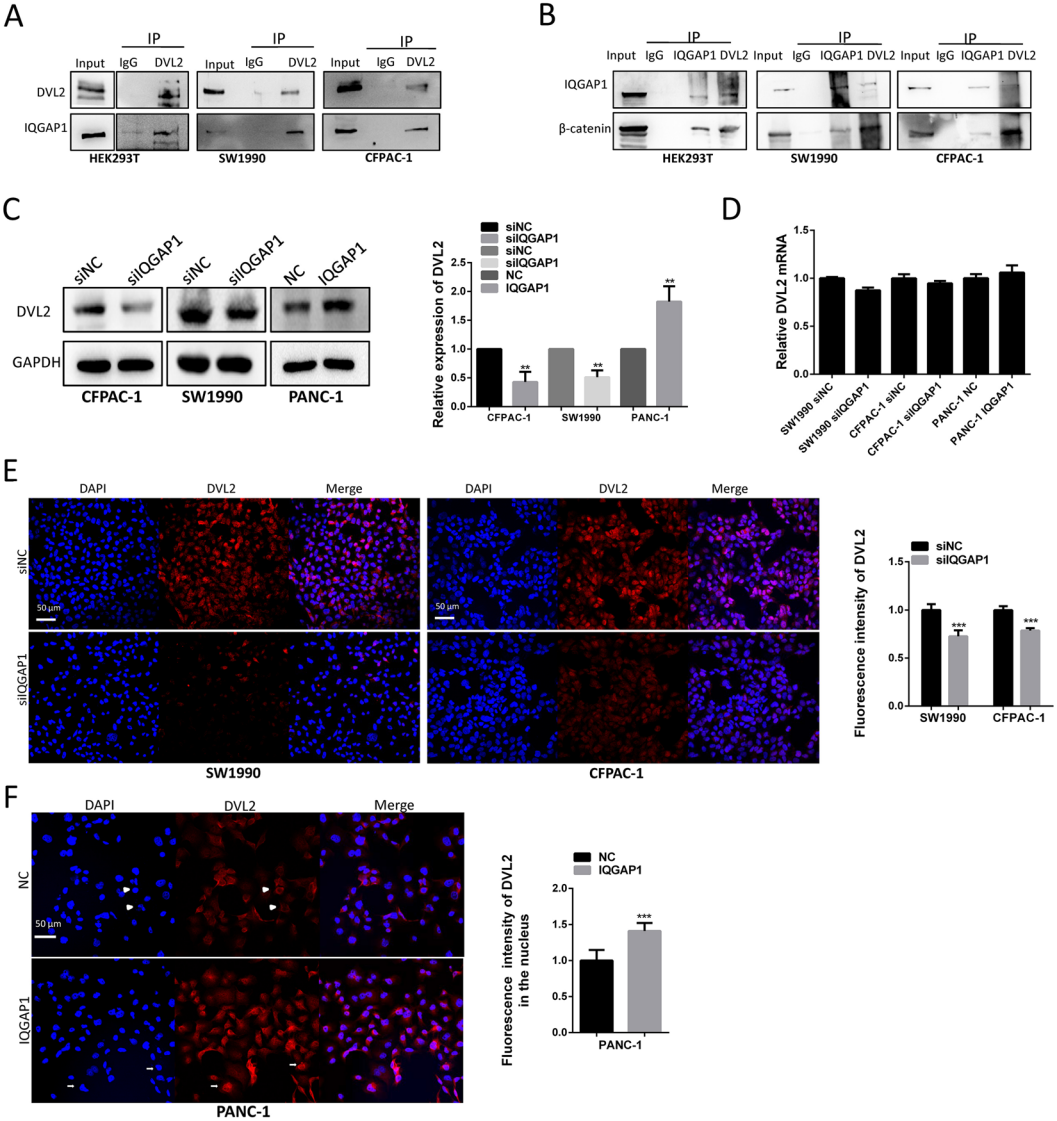
IQGAP1 interacts with DVL2 and translocates DVL2 into the nucleus. (**A**) Co-IP study of non-transfected HEK293T, SW1990 and CFPAC-1 cells confirmed that IQGAP1 and DVL2 can bind with each other. (**B**) Co-IP revealed that both IQGAP1 and DVL2 bind to β-catenin. (**C**) Protein expression of DVL2 was decreased by the suppression of IQGAP1, and overexpression of IQGAP1 had the opposite effect. (**D**) mRNA levels of DVL2 determined by qRT-PCR were not changed by the suppression or overexpression of IQGAP1. (**E**) Immunofluorescence further revealed the change in protein expression and distribution of DVL2 by IQGAP1 knockdown. (**F**) IQGAP1 translocated DVL2 into the nucleus. DVL2 expression in PANC-1 cells transfected with IQGAP1 expression plasmid. Arrows indicate representative cells. Nuclei were visualized by DAPI staining. Uncropped western blots are shown in Supplementary Fig. 2.

During early embryogenesis of Xenopus embryos, the binding of IQGAP1 and DVL2 is required for the canonical Wnt/β-catenin pathway^18^, but the correlation between IQGAP1 and DVL2 in PC cell lines have not been studied. We thus examined whether endogenous IQGAP1 interacts with DVL2 and components of the Wnt/β-catenin pathway. Co-IP experiments verified that IQGAP1 and DVL2 interacted with each other in HEK-293T, SW1990 and CFPAC-1 cells (Fig. 5A). In Xenopus embryos, depletion of IQGAP1 can decrease Wnt-induced nuclear accumulation of β-catenin^19^. Therefore, we questioned whether IQGAP1 can also form a complex with β-catenin in PC cells. Co-IP results demonstrated that endogenous IQGAP1 and β-catenin could interact with each other, suggesting that IQGAP1 could associate with DVL2 and β-catenin to form a complex (Fig. 5B). Therefore, IQGAP1 is involved in the formation of the DVL2 and β-catenin complex in PC cells. Subsequently, we examined the effect of IQGAP1 on DVL2 and Wnt signaling. The protein level of DVL2 decreased when IQGAP1 was knocked down and increased when IQGAP1 was overexpressed (Fig. 5C). However, DVL2 mRNA level was not changed in either IQGAP1-overexpressing cells or knockdown cells (Fig. 5D). Immunofluorescence assays showed that knockdown of IQGAP1 inhibited the overall expression of endogenous DVL2 (Fig. 5E), while overexpression of IQGAP1 promoted the nuclear localization of endogenous DVL2 (Fig. 5F).

### IQGAP1 mediates Wnt/β-catenin signaling through the regulation of expression and interaction of DVL2 and β-catenin

**Figure 6:**
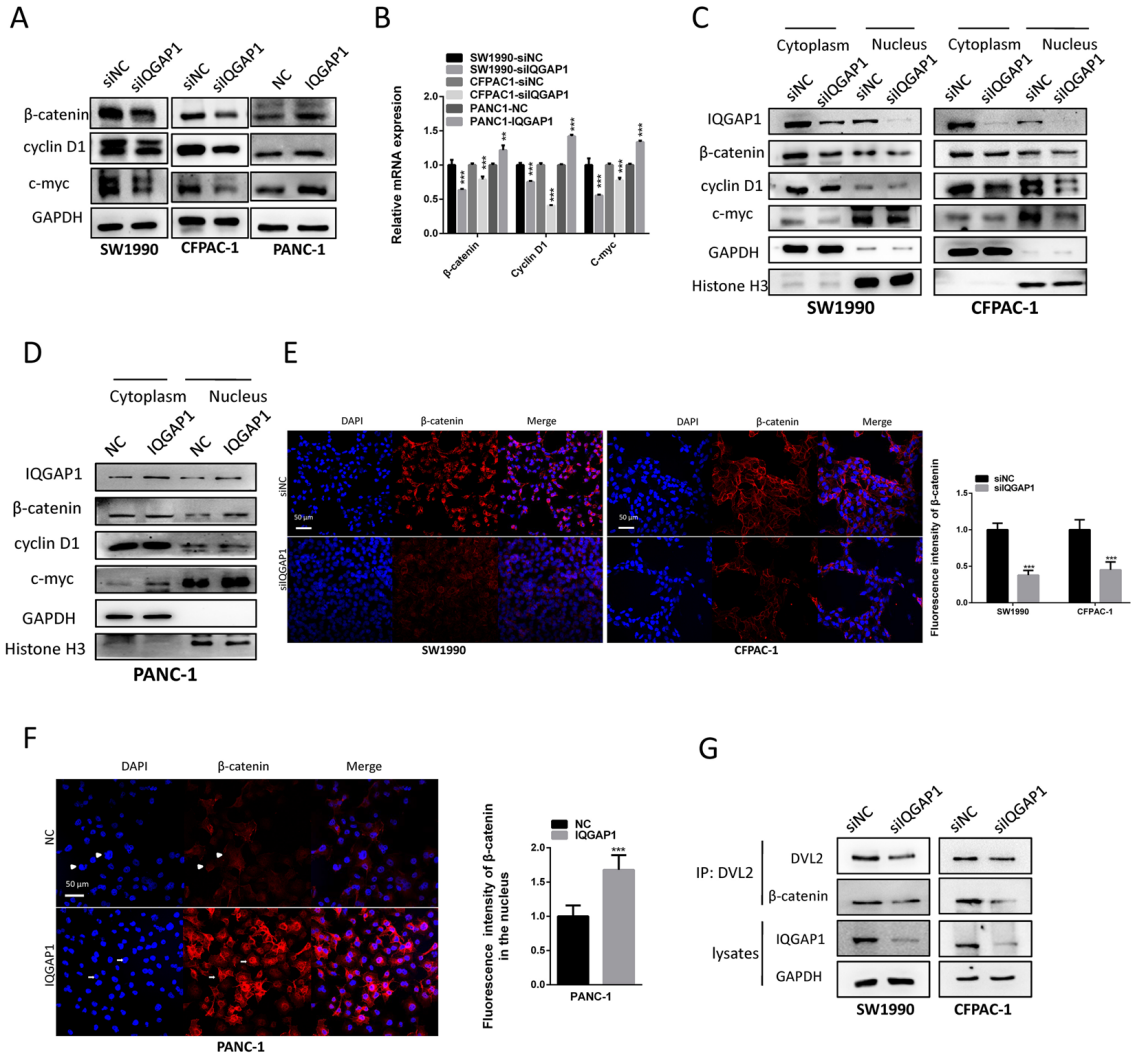
IQGAP1 activates the Wnt/β-catenin signaling pathway and promotes β-catenin translocation into the nucleus. Total β-catenin, cyclin D1and c-myc protein (**A**) and mRNA levels (**B**) were assayed by western blotting and qRT-PCR, respectively, in IQGAP1 knockdown, IQGAP1 overexpression, and control groups. (**C**,**D**) Protein expression of cytoplasmic or nuclear β-catenin and its target genes (cyclin D1 and c-myc) in IQGAP1 knockdown, IQGAP1 overexpression and control groups was assayed by western blotting. (**E**) Immunofluorescence further revealed the change in protein expression and distribution of β-catenin by IQGAP1 knockdown. Nuclei were visualized by DAPI staining. (**F**) IQGAP1 translocates β-catenin into the nucleus. β-catenin expression in PANC-1 cells transfected with IQGAP1 expression plasmid. Arrows indicate representative cells. (**G**) The interaction between β-catenin and DVL2 was determined in SW1990 and CFPAC-1 knockdown cells by Co-IP assay. *P < 0.05, **P < 0.01, and ***P < 0.001. Uncropped western blots are shown in Supplementary Fig. 2.

Given that IQGAP1 binds to both DVL2 and β-catenin, we assessed the effect of IQGAP1 on Wnt/β-catenin activation. Strikingly, inhibition of IQGAP1 downregulated the direct target genes of β-catenin, cyclin D1 and c-myc at both mRNA and protein levels (Fig. 6A,B, Supplementary Fig. 1C). Subcellular fractionation revealed that the downregulation of IQGAP1 expression decreased β-catenin, cyclin D1 and c-myc protein levels in both the cytoplasmic and nuclear fractions (Fig. 6C, Supplementary Fig. 1D,E). Conversely, overexpression of IQGAP1 resulted in increased β-catenin, cyclin D1 and c-myc mRNA and protein levels (Fig. 6A,B, Supplementary Fig. 1C) and enhancement of nuclear accumulation of β-catenin, cyclin D1 and c-myc in PANC-1 cells (Fig. 6D, Supplementary Fig. 1F). Immunofluorescence assays also demonstrated that IQGAP1 regulated both protein level and nuclear translocation of β-catenin, in a similar manner showed in western blotting assays after RNA interference and overexpression of IQGAP1 (Fig. 6E,F).

As IQGAP1 regulated the expression levels and formed complex with DVL2 and β-catenin, it is intriguing to ask whether IQGAP1 plays a role in the interaction between DVL2 and β-catenin. Interestingly, knockdown of IQGAP1 indeed reduced the interaction between DVL2 and β-catenin as demonstrated by Co-IP assay (Fig. 6G). Though expression of IQGAP1 correlates with protein expression levels of β-catenin and DVL2 (Figs 5C, 6A), the ratio between β-catenin and DVL2 in knockdown and overexpression cells showed no difference compared with negative control groups respectively, indicating the expression of IQGAP1 may have altered the levels of β-catenin and DVL2 to a similar degree (Supplementary Fig. 1G). Interestingly, the ratio between co-immunoprecipitated β-catenin and DVL2 decreased in IQGAP1 knockdown cells, indicating IQGAP1 not only regulated protein levels of β-catenin and DVL2 but also mediated their interactions (Supplementary Fig. 1H). Taken together, IQGAP1 may regulate the expression and interaction of β-catenin and DVL2, which subsequently regulates the Wnt/β-catenin signaling pathway.

### IQGAP1 promotes epithelial-mesenchymal transition (EMT) and proliferation through the canonical Wnt signaling pathway by interacting with DVL2

**Figure 7:**
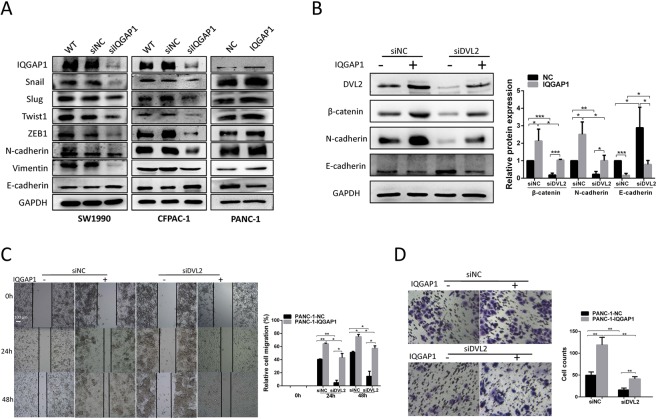
IQGAP1 promotes EMT through the canonical Wnt signaling pathway by interacting with DVL2. (**A**) Western blotting analysis of EMT markers in IQGAP1 knockdown and overexpression PC cells. GAPDH served as a loading control. (**B**) Knockdown of DVL2 impaired the effect of IQGAP1 on EMT. (**C**,**D**) Knockdown of DVL2 impaired the effect of IQGAP1 on wound healing and transwell migration. *P < 0.05, **P < 0.01, and ***P < 0.001. Uncropped western blots are shown in Supplementary Fig. 2.

It is well known that E-cadherin, N-cadherin, and Vimentin are specific markers for the EMT process. We thus tested whether IQGAP1-mediated changes in DVL2 were responsible for changes in EMT. As shown in Fig. 7A, SW1990 and CFPAC-1 cells with IQGAP1 knockdown expressed a higher level of the epithelial marker E-cadherin and lower levels of the mesenchymal markers N-cadherin and Vimentin than did the respective negative control cells. The levels of EMT-activating transcription factors Snail, Slug, Twist1 and ZEB1 were also decreased in SW1990 and CFPAC-1 cells with IQGAP1 knockdown. Conversely, overexpression of IQGAP1 in PANC-1 cells led to the opposite effects (Fig. 7A, Supplementary Fig. 1I–K). Overall, these findings demonstrate that IQGAP1 regulates EMT in PC cells. Because IQGAP1 increases DVL2 protein levels, we questioned whether the expression of EMT markers induced by IQGAP1 is attributable for the enhancement in DVL2 stability. To this end, we co-transfected IQGAP1 expression plasmid and siDVL2 in PANC-1 cells and examined the levels of E-cadherin and N-cadherin. Indeed, siDVL2 reversed the decrease in the expression of E-cadherin and increase in the expression of N-cadherin induced by IQGAP1 overexpression (Fig. 7B), suggesting that EMT regulated by IQGAP1 is mediated by DVL2. Next, we examined the effect of co-transfection of IQGAP1 and siDVL2 on cell migration. In line with the above results, the enhancement in cell migration by the overexpression of IQGAP1 in PANC-1 cells was prevented by siDVL2 transfection as demonstrated using wound healing and transwell migration assays (Fig. 7C,D). Therefore, the downregulation of DVL2 can reverse the EMT process enhanced by IQGAP1.

## Discussion

In this study, we found that the percentage of high expression of IQGAP1 in PDAC tissues was significantly higher than that in adjacent normal tissues, in line with previous results that IQGAP1 is elevated in PC^17^. Furthermore, high IQGAP1 expression was positively correlated with histological differentiation and N stage in PDAC. Some previous studies have reported that the overexpression of IQGAP1 is significantly associated with poor prognosis in advanced colorectal cancer^20^, ovarian carcinoma^21^ and glioma^22^. We revealed that PDAC patients with high IQGAP1 expression had shorter survival times than patients with low IQGAP1 expression and that high IQGAP1 expression was an independent unfavorable prognostic biomarker of OS, suggesting the role of IQGAP1 a potential therapeutic target for PDAC.

IQGAP1 is a scaffold protein that interacts with distinct proteins to regulate cancer cell proliferation and migration^14,23,24^. IQGAP1 has also been reported to play important roles in regulating the microtubule network, actin cytoskeleton and cell adhesion^9^. IQGAP1 has been demonstrated to be regulated by miR-23b cluster, miR-125a-5p^25^, Long noncoding RNA (MIRAT)^26^ and promote RAS-MAP kinase-driven cancer invasion^27^. In addition, a significant increase in angiogenesis by the overexpression of IQGAP1 was found in an *in vivo* mouse model of breast cancer^28^. Moreover, IQGAP1 SUMOylation can stabilize IQGAP1 by reducing protein ubiquitination and thus promote the development of colorectal cancer^29^. It has been reported that IQGAP1 interacts with K-Ras and increases ERK1/2 phosphorylation to modulate K-Ras pathway in PC^30^. Although Jin *et al*. have demonstrated that IQGAP1 expression is positively associated with the degree of metastasis of PC *in vitro*^31^, to fully elucidate the possible functional significance of IQGAP1 in PC cell lines, we performed both gain- and loss-of-function assays. We observed that the knockdown of IQGAP1 in the two cell lines SW1990 and CFPAC-1 not only inhibited cell proliferation *in vitro* but also suppressed tumor growth and metastasis *in vivo*. Conversely, the upregulation of IQGAP1 enhanced proliferation and migration.

Depletion of IQGAP1 can decrease the Wnt-induced nuclear accumulation of β-catenin during early embryogenesis^19^, and IQGAP1 overexpression promotes the transcription and translocation of β-catenin into the nucleus in hepatocellular carcinoma^32^. In contrast, in human colon adenocarcinoma, IQGAP1 knockdown via siRNA cannot alter the expression levels and localization of β-catenin^20^. Our results showed that IQGAP1 knockdown can inhibit canonical Wnt signaling in PC cells as evidenced by decreased levels of both cytoplasmic and nuclear β-catenin, as well as the downstream target genes of β-catenin including cyclin D1 and c-Myc, consistent with previous reports showing that IQGAP1 can regulate β-catenin nuclear localization, which is involved in cell transformation via the Wnt signaling pathway^19^. Our results indicated that the aberrant activation of the canonical Wnt signaling pathway is induced by IQGAP1 upregulation. These results indicate that IQGAP1 is critical for the progression of PC and plays an important role in the aberrant activation of the canonical Wnt/β-catenin pathway.

DVL is critical for protecting β-catenin from degradation in canonical Wnt signaling, where the interaction of DVL with both c-Jun and β-catenin promotes Wnt signaling-stimulated transcription^33^. DVL has three homologues (DVL1, 2 and 3). Some DVL2-associated oncoproteins, such as Forkhead box (FOX) transcription factors (FOXK1 and FOXK2)^34^, ataxia-telangiectasia group D complementing gene (ATDC)^35^, and RAP1B^36^, translocate DVL2 into the nucleus and therefore activate the Wnt/β-catenin signaling pathway. However, some negative regulators such as DVL-DEP domain-Interacting Protein (DDIP) destroy the TCF4/β-catenin complex^37^ and MLLT4 (AF6) independent of the canonical Wnt/β-catenin signaling pathway^38^ and ultimately inhibit cell proliferation. DVL2 has been demonstrated to contribute to PC tumorigenesis by inducing the stabilization of β-catenin^35^. Consistent with the result of other studies, our results revealed that DVL2 can interact with IQGAP1 and that knockdown of DVL2 in PANC-1 cells overexpressing IQGAP1 can reverse the increase in β-catenin levels. By co-IP assays, we confirmed that IQGAP1 forms a complex with DVL2 and β-catenin and may mediate the interaction between DVL2 and β-catenin. We found that IQGAP1 can promote DVL2 nuclear translocation and regulate the protein level but not the mRNA level of DVL2, suggesting that changes in DVL2 stability play an important role in regulating the Wnt/β-catenin signaling pathway. However, the detailed mechanisms remain to be elucidated.

The loss of tumor cell-cell adhesion and gain of tumor invasion enabled by EMT initiates tumor metastasis. E-cadherin-mediated cell-cell adhesion has been proven to be reduced by the overexpression of IQGAP1 via direct interaction with CDC42 and Rac1^39^. IQGAP1 has been documented to be tightly linked with the Wnt/β-catenin signaling pathway^32^ and to be involved in regulation of cell proliferation and EMT^40^. DVL2 is responsible for the regulation of EMT in MCF-7 cells^41^. However, the role of IQGAP1 and DVL2 in the regulation of EMT mediated by Wnt/β-catenin signaling remains obscure. In our study, we provided evidence that IQGAP1 augmented the expression of mesenchymal markers (N-cadherin and Vimentin) and EMT-activating transcription factors (Snail, Slug, Twist1 and ZEB1) while decreasing the expression of the epithelial marker E-cadherin. We also demonstrated that the knockdown of DVL2 inhibited EMT induced by IQGAP1 overexpression. Therefore, we concluded that IQGAP1 may serve an important mediator of EMT through DVL2.

In conclusion, this study indicated that the overexpression of IQGAP1 is associated with unfavorable clinicopathological features and poor survival in patients with PDAC. IQGAP1 can also promote pancreatic tumorigenesis and progression, making it an attractive therapeutic target or prognostic molecular biomarker of PDAC. Our findings show that IQGAP1 regulates Wnt/β-catenin signaling by interacting with DVL2 and β-catenin. Both *in vitro* and *in vivo* results extend our understanding of the underlying molecular mechanism of IQGAP1 in the regulation of EMT and cell proliferation mediated by Wnt/β-catenin signaling. To better reveal the intrinsic mechanism of IQGAP1-induced carcinogenesis, larger populations of patients, longer follow-up and further molecular studies in PC are still needed to support our findings.

## Materials and Methods

### Patients

Human PDAC samples and peritumor samples were obtained from the Department of Hepatobiliary Surgery, Lianyungang Clinical College of Nanjing Medical University. The patients were histopathologically diagnosed with primary PDAC and had not received any anticancer therapy before surgery. PDAC tumors were staged according to the 7^th^ edition of International Union Against Cancer guidelines. Approval from ethics committee of Lianyungang Clinical College of Nanjing Medical University and informed consent from each patient were obtained before enrollment into the study. This study was performed in accordance with the Declaration of Helsinki.

### Stable cell line construction

Based on the silencing effect of small interfering RNAs (siRNAs) against IQGAP1, we chose sense 5′-GCGACAAAGUCCUGAACAU-3′ and antisense 5′-AUGUUCAGGACUUUGUCGC-3′ to be inserted into the lentiviral expression vector hU6-MCS-Ubiquitin-EGFP-IRES-puromycin (Shanghai GenePharma, Co., Ltd., Shanghai, China). GFP in all lentiviral vectors was used to monitor transfection efficiency. SW1990 cells were seeded in 6-well plates and transfected with IQGAP1-targeting shRNA and negative control shRNA. Forty-eight hours after infection, the cells were treated with puromycin (1 μg/ml) for approximately two weeks to generate stable transfected cells.

### Xenograft tumorigenicity assays

All male BALB/c nude mice, aged 4–6 weeks, used in this study were purchased from Model Animal Research Center at Nanjing University (Nanjing, China). All the animal protocols were reviewed and approved by the Animal Care Committee of Nanjing University in accordance with the guidelines of the Institutional Animal Care and Use Committee. To assess tumor growth, we subcutaneously implanted SW1990 cells (5 × 10^6^) into the mice (6 mice per group). The mice were randomly divided into two groups after 24 days of tumor implantation. Tumor volume was calculated by the following formula: volume = (length × width^2^)/2. For metastasis study, SW1990 cells (1 × 10^6^) in 50 μL phosphate-buffered saline were orthotopically injected into the major pancreatic duct (8 mice per group). Three months after injection, the mice were euthanized, and suspected metastatic tissues were subjected to hematoxylin and eosin (H&E) staining for the pathologic examination of metastatic nodules.

### Others

For reagents, cell lines and cell culture, tissue microarray (TMA) construction, immunohistochemistry and scoring, small interfering RNA and plasmid vector-related assays, quantitative real-time reverse transcription polymerase chain reaction, western blotting, CCK-8 assay, colony formation assay, wound healing assay, transwell migration assay, co-immunoprecipitation (co-IP) experiments, and immunofluorescence imaging, please see Supplemental File.

### Statistical analysis

Associations of categorical data between IQGAP1 expression and clinicopathological features were assessed using the chi-square test and Fisher’s exact test. Possible differences in overall survival (OS) were examined by the Kaplan-Meier analysis and log-rank test. Prognostic factors associated with OS in univariate analysis were evaluated using Cox regression models for multivariate analysis. Student’s t-tests were used to determine the differences between two independent groups, and the results were expressed as the mean ± SD. All statistical analyses were performed using SPSS 23 statistical software (SPSS Inc, Chicago, IL, USA). *P* value < 0.05 was considered statistically significant.

## Supplementary information

Supplemental Information

## Data Availability

All data generated or analyzed during this study are available from the corresponding author on reasonable request.
