# Supplementary material for: IQGAP1 promotes pancreatic cancer progression and epithelial-mesenchymal transition (EMT) through Wnt/β-catenin signaling
Source: Sci Rep. 2019 May 17;9:7539. doi: 10.1038/s41598-019-44048-y (PMC6525164; doi:10.1038/s41598-019-44048-y)

IQGAP1 promotes pancreatic cancer progression and epithelial-mesenchymal transition (EMT) through Wnt/β-catenin signaling

Wei Hu[1, 2], Zhongxia Wang[2], Shan Zhang[2], Xian Lu[2], Junyi Wu[2], Kuanyong Yu[2], Anlai Ji[2], Wei Lu[4], Zhong Wang[1], Junhua Wu[3] and Chunping Jiang[2]

[1]Department of Hepatobiliary Surgery, Lianyungang Clinical College of Nanjing Medical University, Lianyungang, 222001 Jiangsu, China.

[2]Department of Hepatobiliary Surgery, Drum Tower Clinical College of Nanjing Medical University, Nanjing, 210008 Jiangsu, China.

[3]Jiangsu Key Laboratory of Molecular Medicine, Medical School, Nanjing University, Nanjing, 210093 Jiangsu, China.

[4]Jiangsu Province Hospital of Traditional Chinese Medicine, Nanjing, 210029 Jiangsu, China.

Wei Hu, Zhongxia Wang and Shan Zhang contributed equally to this work.

Correspondence and requests for materials should be addressed to C.P.J. (email: chunpingjiang@163.com), J.H.W. (email: wujunhua@nju.edu.cn) or Z.W. (email: 18961326366@189.cn).

Supplemental Methods

Reagents

Anti-human β-catenin, cyclin D1, DVL2, Vimentin, N-cadherin, E-cadherin, snail, slug, twist1 and ZEB1antibodies were purchased from Cell Signaling Technology (Beverly, MA, USA). Anti-human IQGAP1 and c-myc antibody were obtained from Abcam Company (Cambridge, MA, USA). The control antibody (anti-GAPDH and anti-Histone H3) was purchased from Bioworld Technology, Inc. (St. Louis Park, MN, USA) and Proteintech (Chicago, IL, USA), respectively. SiRNAs for IQGAP1 and DVL2 and respective negative controls were purchased from GenePharma (Shanghai GenePharma Co., Ltd., Shanghai, China). Cell counting kit-8 (CCK-8) was purchased from Dojindo. Trizol reagent and PrimeScript RT Master Mix (Perfect Real Time) were both obtained from TaKaRa (Takara Bio Inc., Naha, Japan).

Cell lines and cell culture

HEK293T cells, pancreatic cancer cell lines SW1990, BxPc-3, Capan-2, CFPAC-1, PANC-1 and normal human pancreatic ductal cell line hTERT-HPNE were purchased from the American Type Culture Collection (Rockville, MD, USA). HEK293T cells and Pancreatic cancer cell lines were cultured in Dulbecco's Modified Eagle's Medium (DMEM, Gibco, Gaithersburg, MD, USA) supplemented with 10% fetal bovine serum (FBS), penicillin (100 U/ml) and streptomycin (100 ug/ml)) at 37°C in 5% CO2. hTERT HPNE was maintained in medium containing3 vol of glucose-free DMEM (Sigma Cat#. D-5030, with additional 2 mM L-glutamine and 1.5 g/L sodium bicarbonate), 1 vol of Medium M3 Base (InCell Cat# M300F- 500, San Antonio, TX, USA), 5% FBS, 10 ng/ml human recombinant EGF, 5.5 mM D-glucose, and 750 ng/ml puromycin at 37°C under air with 5% CO2.

Tissue microarray construction

Tissue microarrays (TMAs) were obtained with 87 PDAC tissues and 69 corresponding pericancerous tissues. Well-documented clinicopathological information was given with the TMAs, such as patients' age, gender, pathological grade, tumor location and size, perineural invasion, lymph node metastasis, tumor stage, and the follow-up data. Total 87 patients included 54 males and 33 females, aged from 34 to 85 years (mean age, 61 years). There are still 23 patients alive till the end of the follow-up. The definition of follow-up period was the interval from the date of the operation to the date of either patient death or the final follow-up. The follow-up time ranged from 0 to 87 months with a median time of 10 months. The TMAs were performed for immunostaining by using anti-IQGAP1

antibodies.

Immunohistochemistry and scoring

The IQGAP1 protein levels in tumor and peri-tumor tissues were detected by IHC following published protocols[1]. Tissue was fixed in formaldehyde and a heat mediated antigen retrieval step was performed by using citrate. Samples were incubated with a rabbit polyclonal antibody against IQGAP1 (1:400 dilutions; no. ab86064, Abcam Inc., USA) overnight at 4°C and then with a horseradish peroxidase conjugated secondary antibody. The IQGAP1 protein level was visualized by 3, 3'-diaminobenzidine (DAB) staining at room temperature for 5 min, after which the sections were counterstained with hematoxylin. All stained sections were independently scored by two pathologists with no knowledge of the clinical data. The ratio of positive tumor cells was evaluated quantitatively as previously described: 0, staining of <10%; 1+, staining of 10-25%; 2+, staining of 25-50%; 3+, staining of 50–75%; and 4+, staining of >75%[2]. Staining intensity was scored from 0 (none) to 3 (strong staining). The staining index (SI) was calculated as the ratio of positive tumor cells multiplied by the staining intensity. An optimal cutoff value was identified as follows: the SI score of >4 was used to define PDAC as high expression ($IQGAP1^H$), whereas the SI score of ≤4 as low expression ($IQGAP1^L$).

Small interfering RNA and plasmid vector related assays

The pcDNA3.1-IQGAP1 plasmid was synthesized by Genechem (Shanghai Genechem Co., Ltd., Shanghai, China) andtransfected by using Lipofectamine 3000 (Thermo Fisher Scientific, MA, USA) reagent according to the manufacturer's instructions.. The pcDNA3.1 empty vector was used as a negative control. Si-RNAs were designed and synthesized by GenePharma (Shanghai GenePharma Co., Ltd., Shanghai, China). The transfection was conducted by using Lipofectamine® RNAiMAX (Thermo Fisher Scientific, MA, USA) following the manufacturer's instructions. Following transfection for 48-72 h, cells were collected for subsequent experiments.

Target sequences for siRNA and shRNA shown in this study.

	Sense(5'-3')
IQGAP1 siRNA1#	GGAUGAAGCCGCAUUACAU
IQGAP1 siRNA2#	GCGACAAAGUCCUGAACAU
DVL2-siRNA	UCCACAAUGUCUCUCAAUA
NC-siRNA	UUCUCCGAACGUGUCACGU
IQGAP1-shRNA	GCGACAAAGTCCTGAACAT
NC-shRNA	TTCTCCGAACGTGTCACGT

Quantitative real time reverse transcription polymerase chain reaction

Total RNA was extracted from different cell lines using Trizol reagent and reverse-transcribed into cDNA with PrimeScript RT Master Mix as previously described[3]. The qRT-PCR amplification was performed using Power SYBR Green PCR master mix (Takara Bio Inc., Naha, Japan). All the experiments mentioned here were performed according to the manufacturer's instructions. The sequences of the primers are listed in following Table. Each experiment was performed at least three times. Relative quantification of mRNA expression within the samples was calculated using the ΔΔCt method.

Primers for real-time RT-PCR used in this study.

	Primer sequences (5'-3')
IQGAP1	5′ GGTGGAATCTGGCAGTCCAA 3′
	5′ ACAAGCCAACATCAGGGGAG 3′
β-catenin	5′ CACAAGCAGAGTGCTGAAGGTG 3′
	5′ GATTCCTGAGAGTCCAAAGACAG 3′
cyclin D1	5′ TGGAGGTCTGCGAGGAACA 3′
	5′ TTCATCTTAGAGGCCACGAACA 3′
c-myc	5′ GCCACGTCTCCACACATCAG 3′
	5′ TGGTGCATTTTCGGTTGTTG 3′
DVL2	5′ CGTCACAGATTCCACAATGTCT 3′
	5′ TCGTTGCTCATGTTCTCAAAGT 3′
GAPDH	5′CCATGTTCGTCATGGGTGTGAACCA 3′
	5′GCCAGTAGAGGCAGGGATGATGTTC 3′

Western Blot assay

Total protein was extracted by using a NP40 lysis buffer containing 150 mM NaCl, 1% Nonidet P-40, 50 mM Tris/HCl (pH 7.4) supplemented with protease and phosphatase inhibitors (Thermo Fisher Scientific, MA, USA). After quantified through a BCA protein assay kit (Thermo Fisher Scientific, MA, USA), the proteins were fractionated by SDS-PAGE electrophoresis. Western blot analysis was performed by using standard methods. Nuclear and cytoplasmic proteins were extracted using NE-PER Nuclear and Cytoplasmic Extraction Reagents (Beyotime Biotechnology, Haimen, China) according to the manufacturer's protocol.

CCK-8 assay

Cells transfected with siRNA for IQGAP1 and negative controls were plated in 96-well plates at a density of 2.5×10^3 cells/well. 100 μL complete medium containing 10 μL CCK-8 reagent (Dojindo Laboratories, Kumamoto, Japan) was added to each well at different time points (24 h, 48 h, 72 h, 96 h, and 120 h). The plates were incubated in dark at 37°C for 2 h and analyzed by absorbance at 450 nm, as previously described[4]. At least three wells were

assessed for each group.

Colony formation assay

Different cell lines were seeded in 6-well plates (5×10^2 cells/well) and incubated at 37°C with 5% CO2 for 10 days. The culture medium was changed every 3 days. Surviving colonies were stained with crystal violet and then counted to evaluate cell proliferation.

Wound healing assay

Cells were seeded at a density of 1×10^5 per well in Wound Healing Culture Inserts (EMD Millipore, Billerica, MA, Germany) fixed solidly in 24-well plates. When cells were adhere-wall and reached 95% confluence, the culture Insert was removed. Cells were washed with PBS and cultured in serum-free DMEM. Then photographs were recorded at 0, 24 and 48 hours.

Transwell migration assay

The transwell assay was performed by using Transwell inserts (EMD Millipore, Billerica, MA, Germany). In brief, 2×10^4 cells were added into the upper chamber (pore size, 8 μm; Millipore, Germany) containing 200 μL serum-free medium. The bottom chamber was filled with 600 μL culture medium containing 10% FBS. Nonmigrating cells on the upper surface were removed with a cotton swab. The filters were then fixed, stained and counted under a microscope (×200) in three randomly chosen fields.

Coimmunoprecipitation Experiments

HEK 293T cells, SW1990 and CFPAC-1 cells were harvested to dissolve with NP40 lysis buffer on ice for 5 min. Cell lysates were then centrifuged at 12000 g for 10 min. The supernatant was incubated overnight at 4°C with anti-IQGAP1 antibody (2-5 μg/mg of lysate), anti-DVL2 (1:100) and anti-β-catenin antibody (1:50), respectively. And rabbit IgG (A7016 Beyotime, 1μg) was taken as a control. Protein A sepharose beads (Beyotime, Haimen, China) were added and rotated for 3 h at 4°C. The precipitates were washed five times with NP40 buffer, boiled for 5 min and then subjected to western blotting analysis.

Immunofluorescence imaging

Cells grown on coverslips were fixed with 4% paraformaldehyde for 15 min at room temperature and incubated overnight with indicated primary antibodies at 4°C. After that, the cells were incubated with Alexa Fluor-conjugated secondary antibodies (Thermo Fisher Scientific, MA, USA), and the nucleus was counterstained with DAPI (Thermo

Fisher Scientific, MA, USA). Fluorescence was photographed by confocal laser microscopy (FV3000, Olympus).

References:

1. Song, K. *et al.* Transforming Growth Factor TGFbeta Increases Levels of Microtubule-Associated Protein MAP1S and Autophagy Flux in Pancreatic Ductal Adenocarcinomas. *PLOS ONE* **10**, e143150 (2015).
2. Hayashi, H. *et al.* Overexpression of IQGAP1 in advanced colorectal cancer correlates with poor prognosis-critical role in tumor invasion. *INT J CANCER* **126**, 2563-2574 (2010).
3. Chen, A. *et al.* Oncolytic measles virus enhances antitumour responses of adoptive CD8(+)NKG2D(+) cells in hepatocellular carcinoma treatment. *Sci Rep* **7**, 5170 (2017).
4. Peng, Y.P. *et al.* PEG10 overexpression induced by E2F-1 promotes cell proliferation, migration, and invasion in pancreatic cancer. *J Exp Clin Cancer Res* **36**, 30 (2017).

Supplementary Table S1 Univariate and multivariate survival analyses between clinicopathological characteristics and IQGAP1 expression in patients with PDAC

Variables	mOS 95% CI, months	Univariate			Multivariate		
		HR	95% CI	*P**	HR	95% CI	*P**
Sex							
Male	10.0 (7.600-12.400)	1.082	0.649-1.804	0.762			
Female	11.0 (2.030-19.970)	1					
Age							
≤61	13.0 (6.499-19.501)	0.794	0.486-1.298	0.358			
>61	10.0 (7.886-12.114)	1					
Tumor location							
Pancreatic head	11.0 (8.068-13.932)	0.847	0.513-1.400	0.518			
Non-head	10.0 (7.273-12.727)	1					
Histological differentiation							
Well-moderate	12.0 (7.579-16.421)	1			1		
Poor	8.0 (5.437-10.563)	1.741	1.006-3.011	0.047	1.969	1.107-3.500	0.021
Tumor size (cm)							
<4	10.0 (6.263-13.737)	1.159	0.683-1.968	0.585			
≥4	11.0 (8.648-13.352)	1					
TNM stage							
III+IV	23.0	1					
I+II	10.0 (8.296-11.704)	1.019	0.141-7.375	0.985			
T classification							
T1/T2	11.0 (9.275-12.725)	0.917	0.506-1.659	0.774			
T3	10.0 (0.000-21.375)	1					
N classification							
N0	33.0 (14.801-51.199)	1			1		
N1	10.0 (8.988-11.012)	2.577	1.533-4.332	<0.001	2.786	1.617-4.800	<0.001
M classification							
M0	10.0 (8.296-11.704)	1					
M1	23.0	0.981	0.136-7.101	0.985			
Perineural invasion							
Present	10.0 (8.267-11.733)	1					
Absent	11.0 (6.962-15.038)	0.814	0.497-1.335	0.416			
IQGAP1 expression							
High	10.0 (8.325-11.675)	2.270	1.295-3.978	0.004	1.880	1.049-3.367	0.034
Low	33.0 (0.085-65.915)	1			1		

mOS median overall survival, HR hazard ratio, CI confidence interval
*$P<0.05$

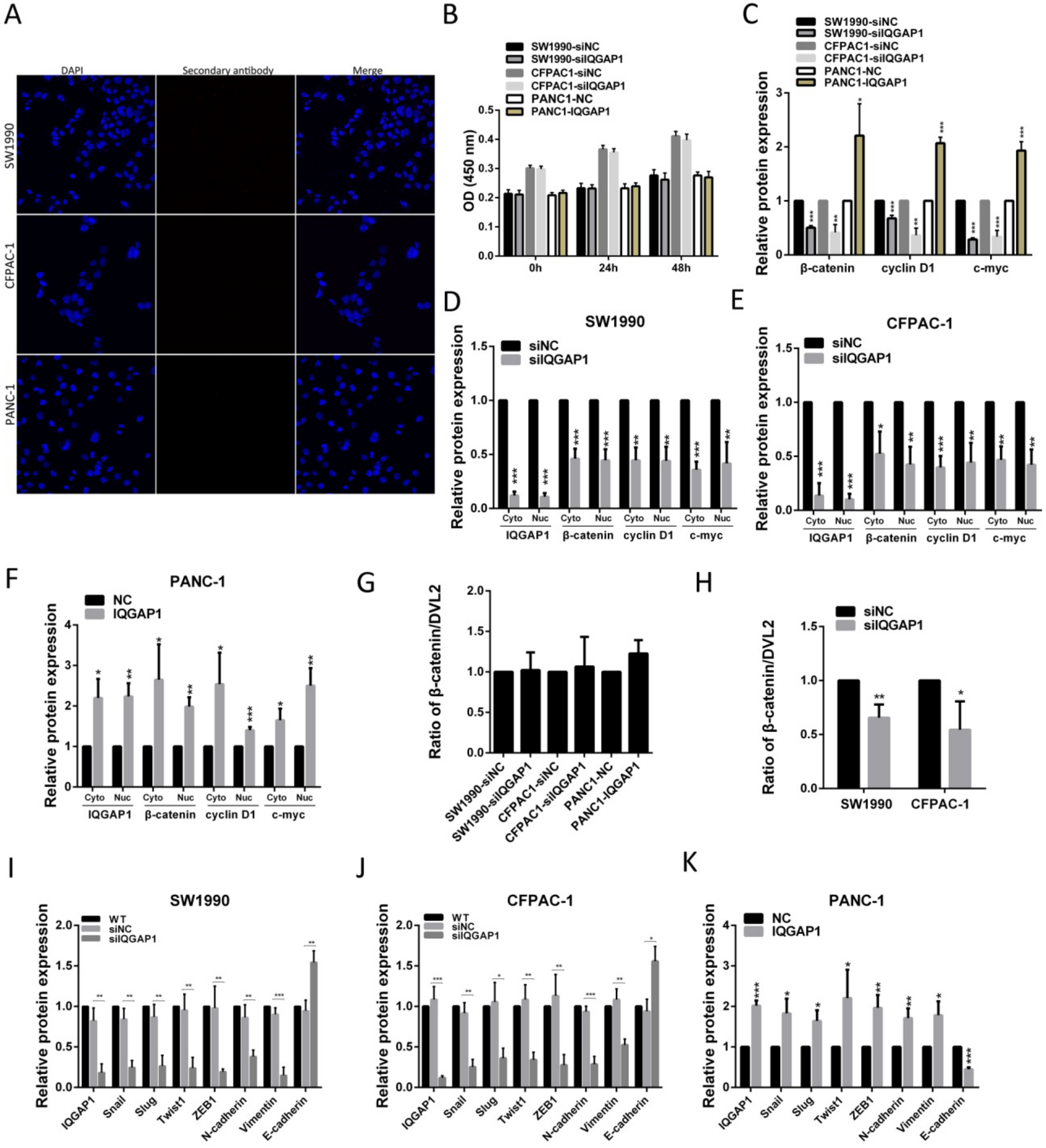

Supplementary Figure 1.

(**A**) Secondary antibody only staining was used as negative control for immunofluorescence assay. Supplementary data for Fig. 2E.

(**B**) CCK-8 cell proliferation assay mimicking culture condition during wound healing assay showed no significant difference between groups with different transfection conditions.

(**C**) Densitometry analysis of β-catenin, cyclin D1 and c-myc in cells transfected with scrambled RNA (siNC), IQGAP1 siRNA (siIQGAP1), negative control vector (NC) and IQGAP1 overexpressing vector (IQGAP1). Supplementary data for Fig. 6A.

(**D, E**) Densitometry analysis of cytoplasmic and nuclear IQGAP1, β-catenin and downstream target genes (cyclin D1 and c-myc) in cells transfected with scrambled RNA (siNC) and IQGAP1 siRNA (siIQGAP1). Supplementary data for Fig. 6C.

(**F**) Densitometry analysis of cytoplasmic and nuclear IQGAP1, β-catenin and downstream target genes (cyclin D1 and c-myc) in cells transfected with negative control vector (NC) and IQGAP1 overexpressing vector (IQGAP1). Supplementary data for Fig. 6D.

(**G**) Densitometry showed ratios between β-catenin and DVL2 in cells transfected with scrambled RNA (siNC), IQGAP1 siRNA (siIQGAP1), negative control vector (NC) and IQGAP1 overexpressing vector (IQGAP1) in western blotting assays. Supplementary data for Fig. 5C and 6A.

(**H**) Densitometry showed ratios between β-catenin and DVL2 in cells transfected with scrambled RNA (siNC) and IQGAP1 siRNA (siIQGAP1) in co-immunoprecipitation assay. Supplementary data for Fig. 6G.

(**I, J, K**) Densitometry analysis of EMT markers in wild type cells (WT) and cells transfected with scrambled RNA (siNC), IQGAP1 siRNA (siIQGAP1), negative control vector (NC) and IQGAP1 overexpressing vector (IQGAP1). Supplementary data for Fig. 7A.

Supplementary Figure 2: Unprocessed scans of original western blots used in the main figures

Blots for Figure 1C

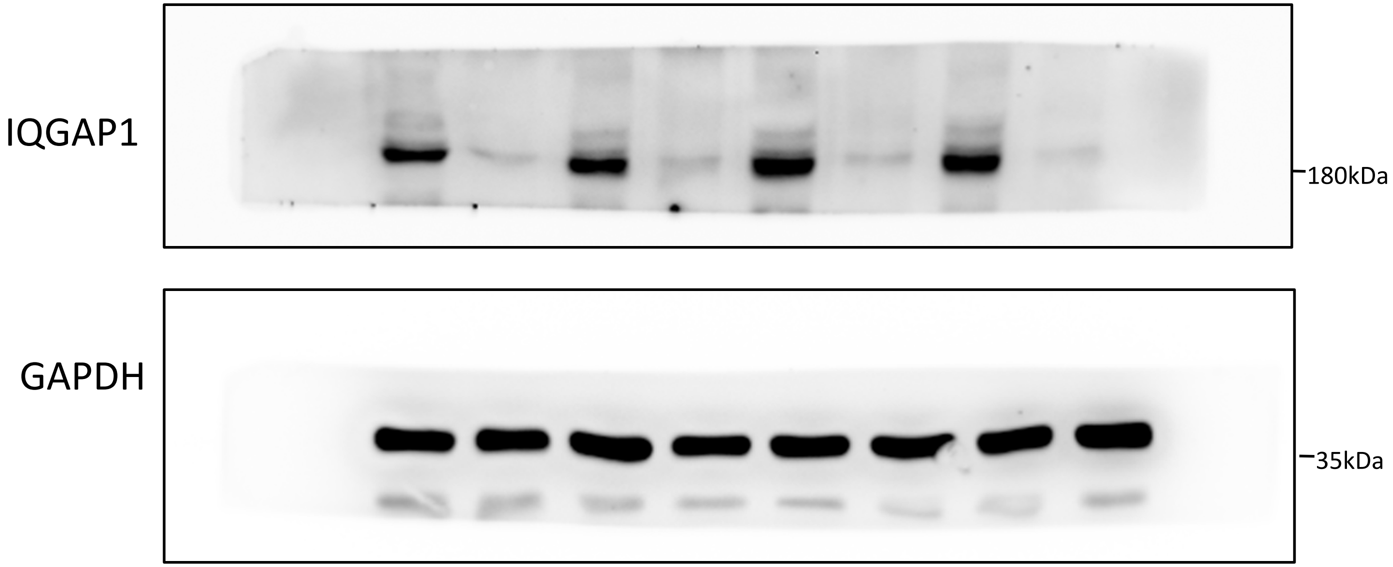

Blots for Figure 2

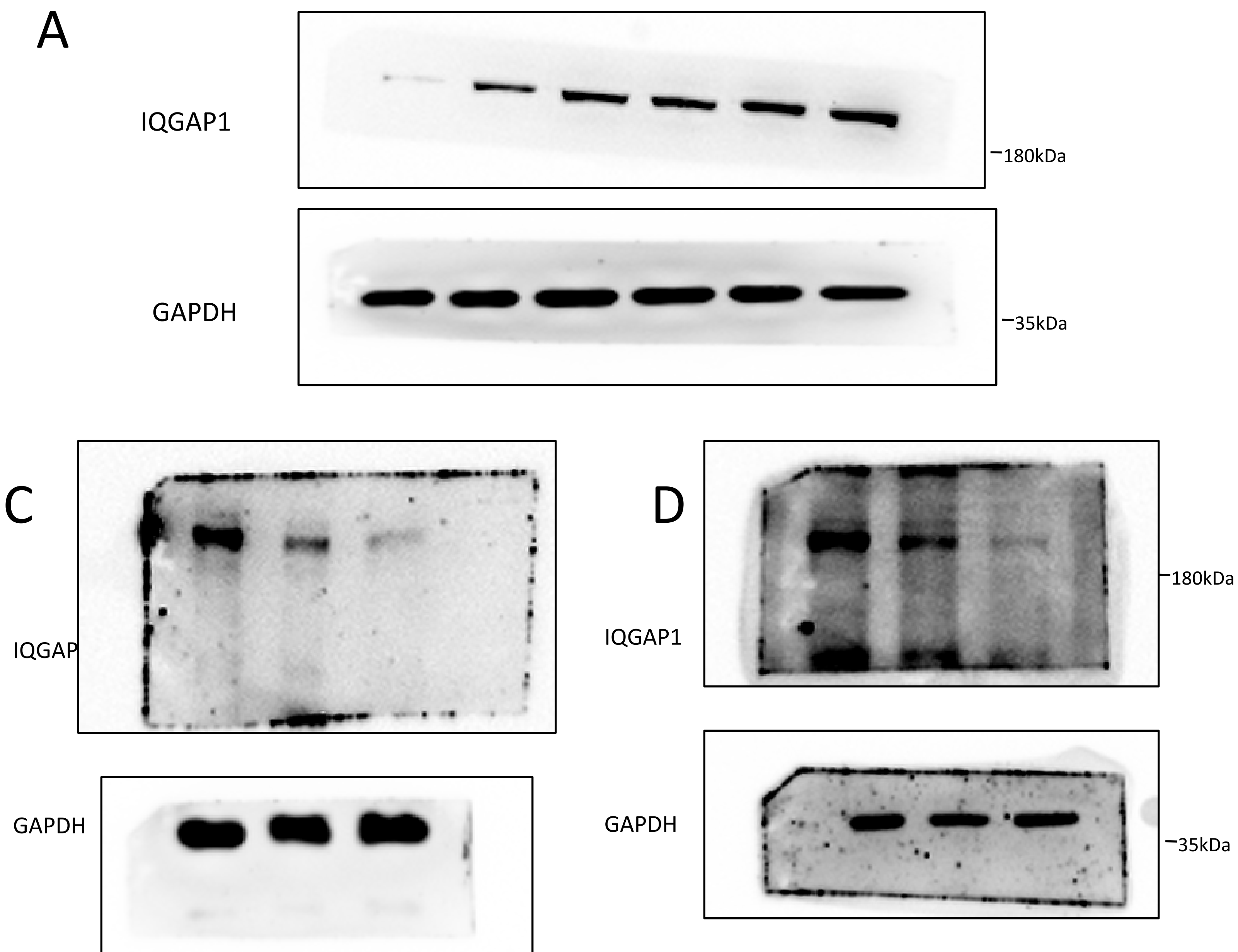

Blots for Figure 3E

IQGAP1

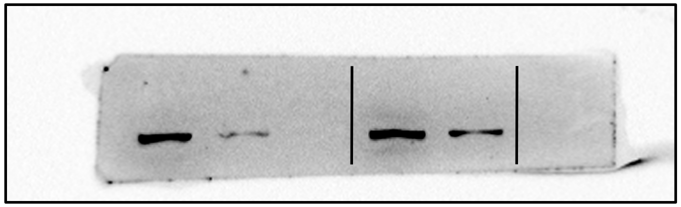

—180kDa

GAPDH

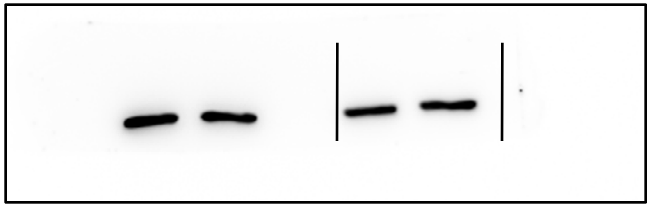

—35kDa

Blots for Figure 4A

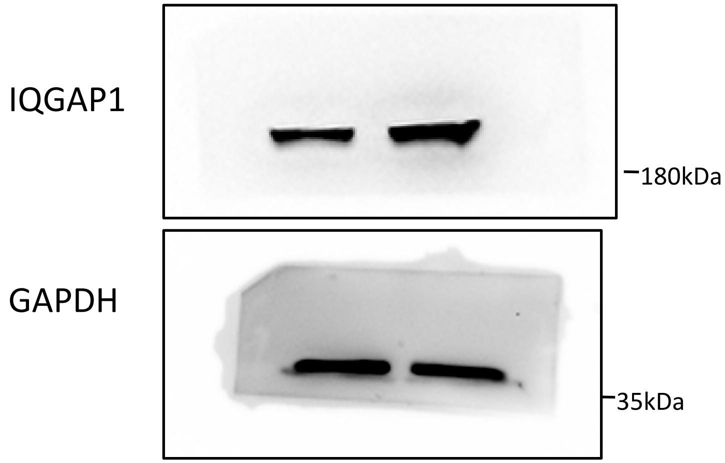

Blots for Figure 5

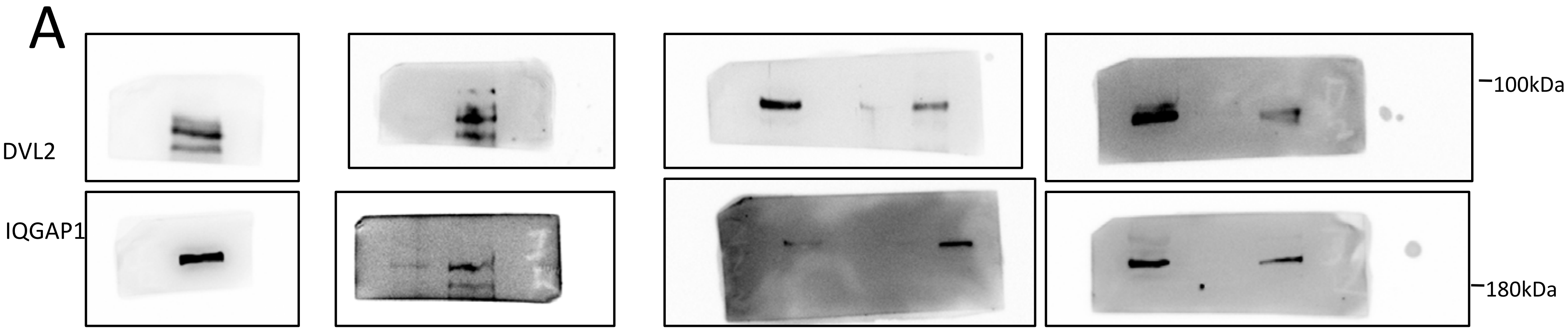

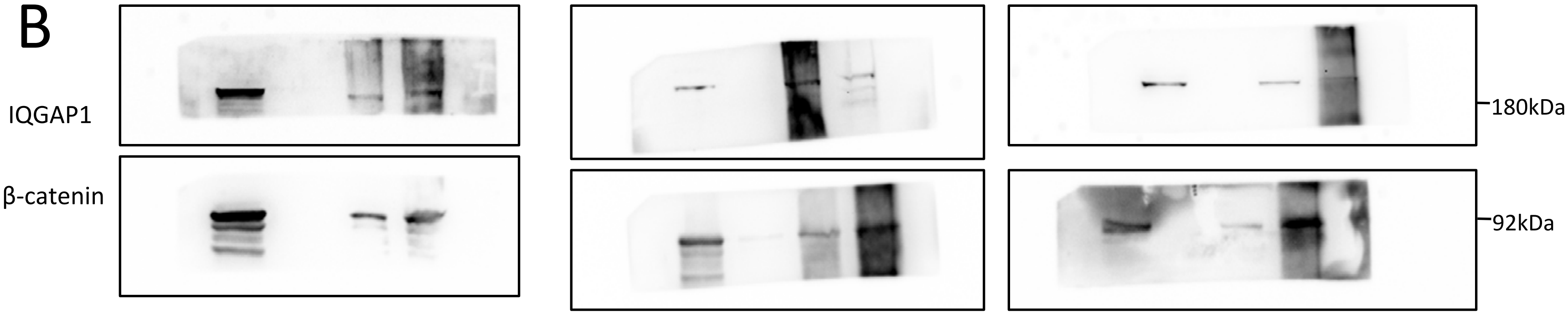

Blots for Figure 5C

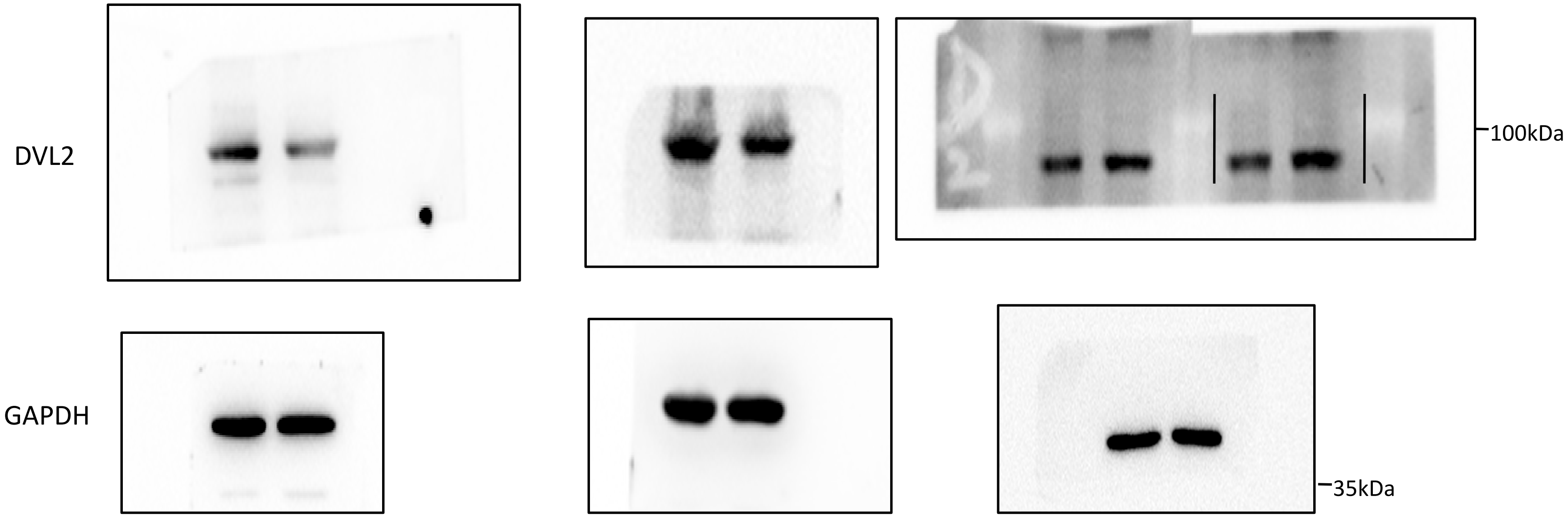

Blots for Figure 6A

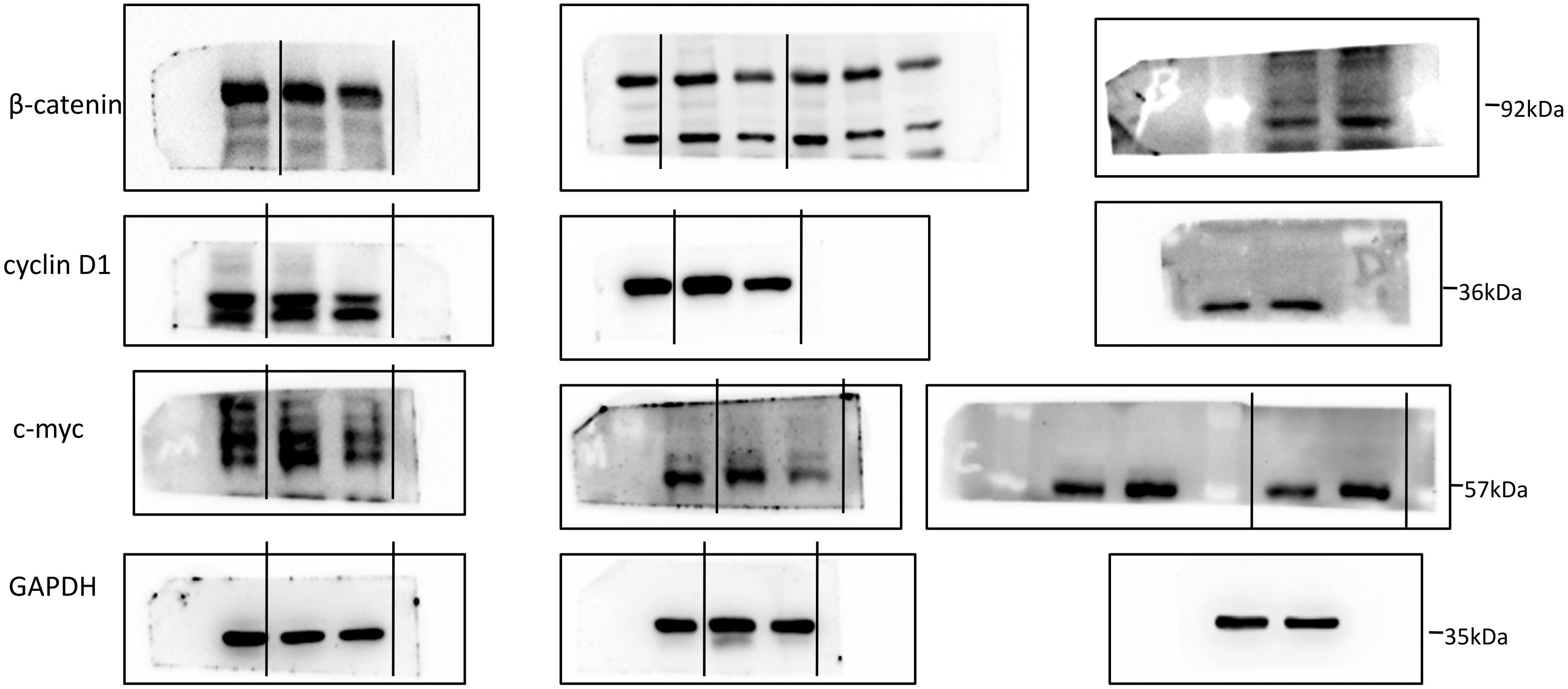

Blots for Figure 6C

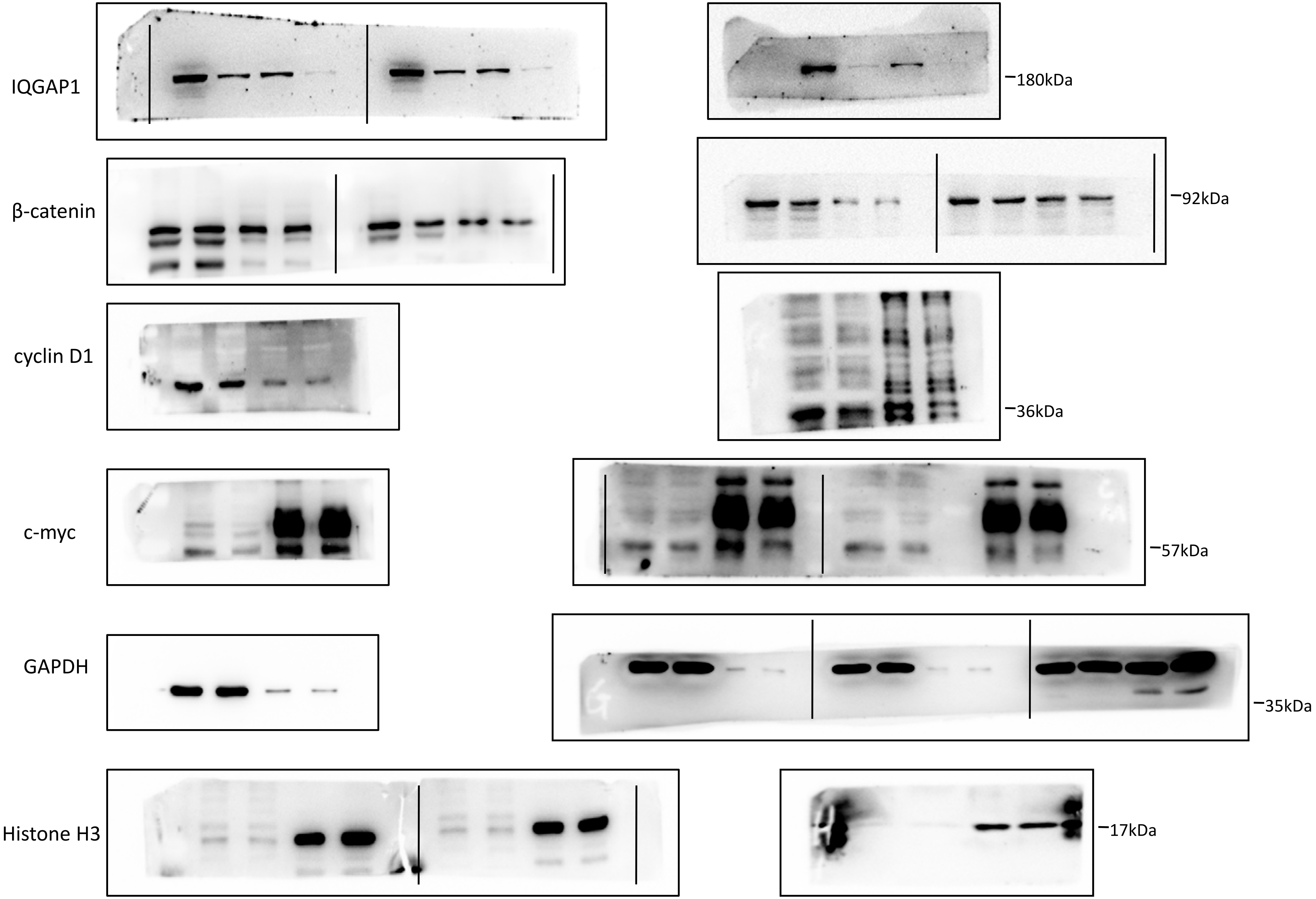

Blots for Figure 6D

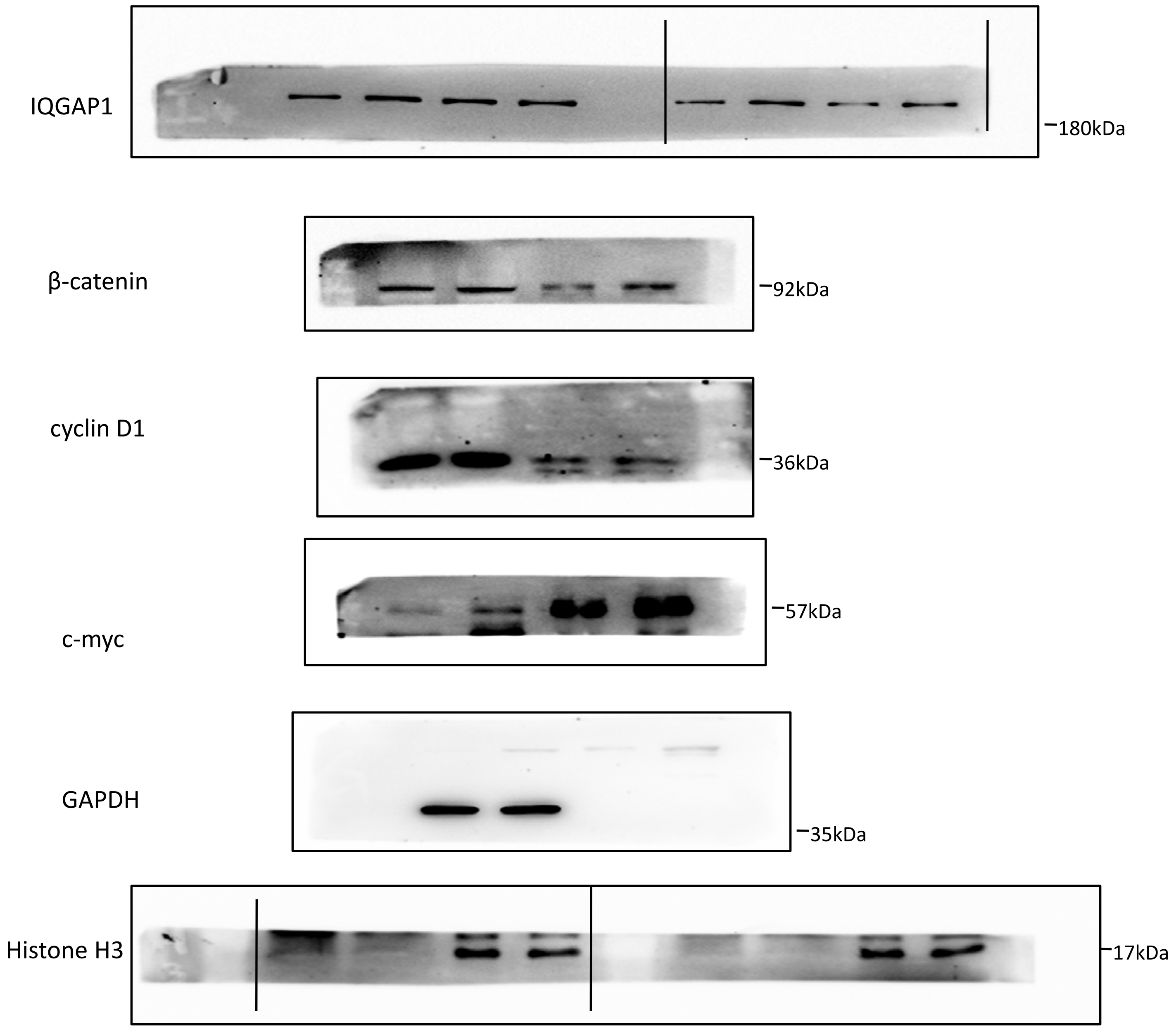

Blots for Figure 6G

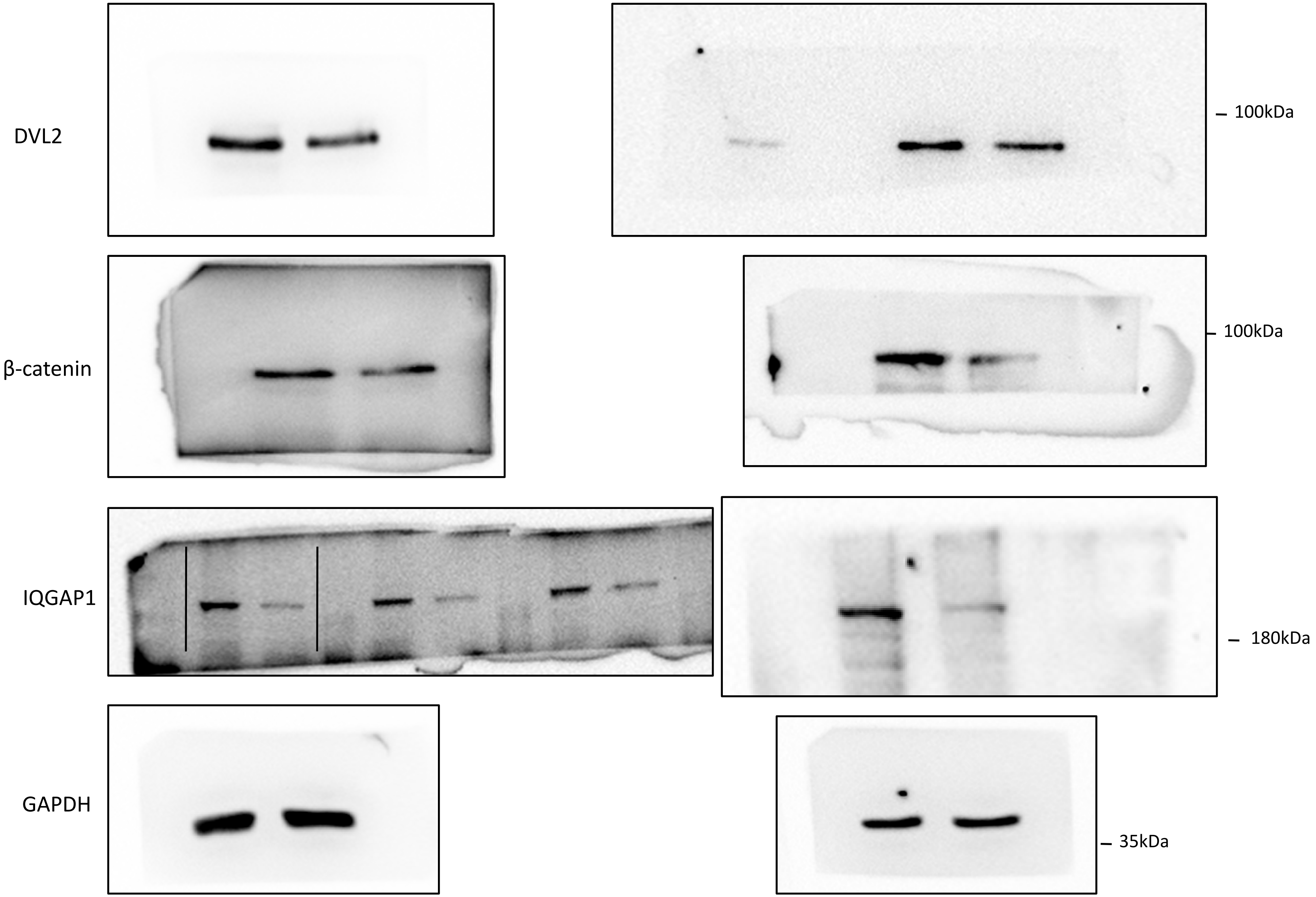

Blots for Figure 7A

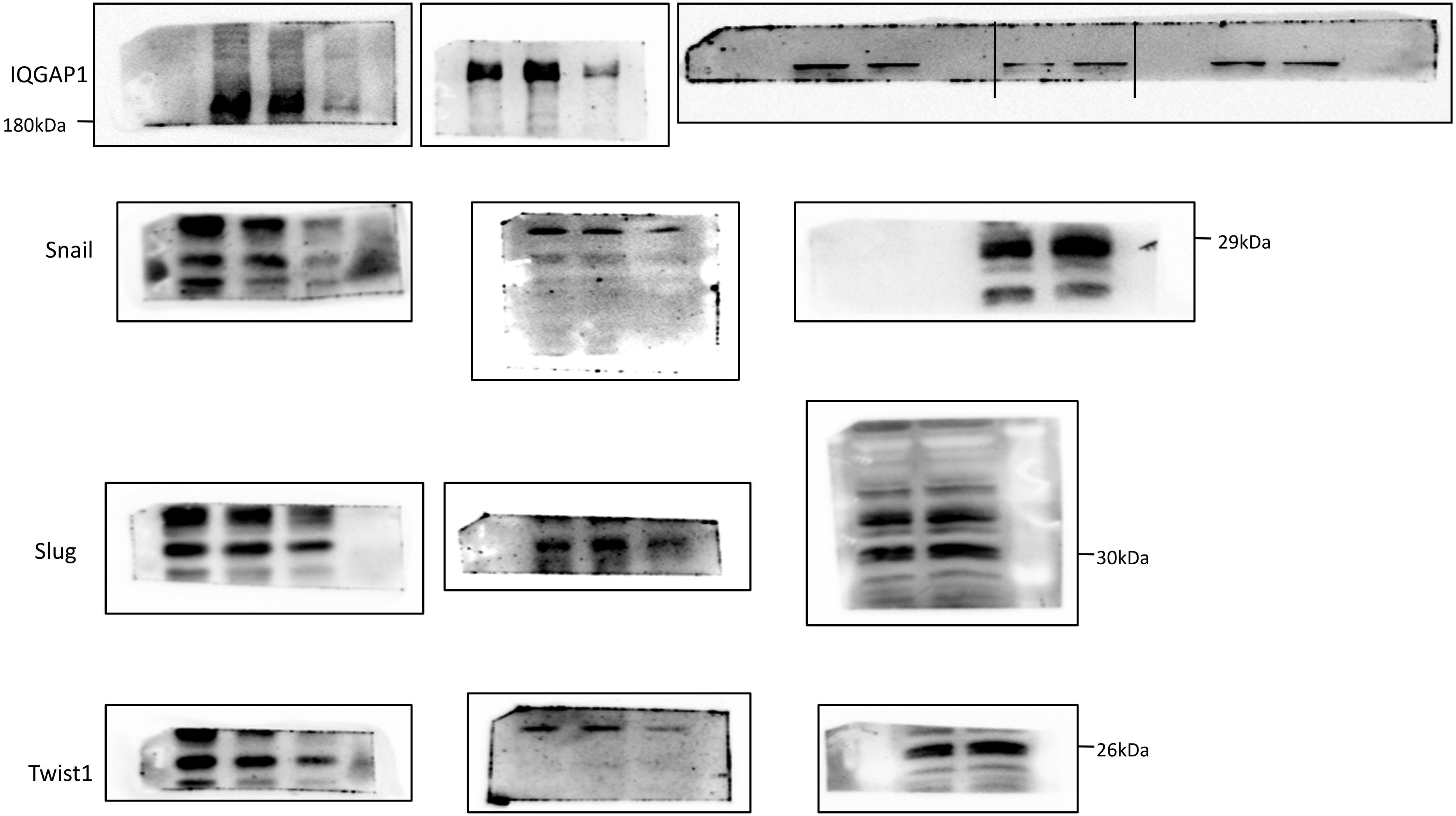

Blots for Figure 7A

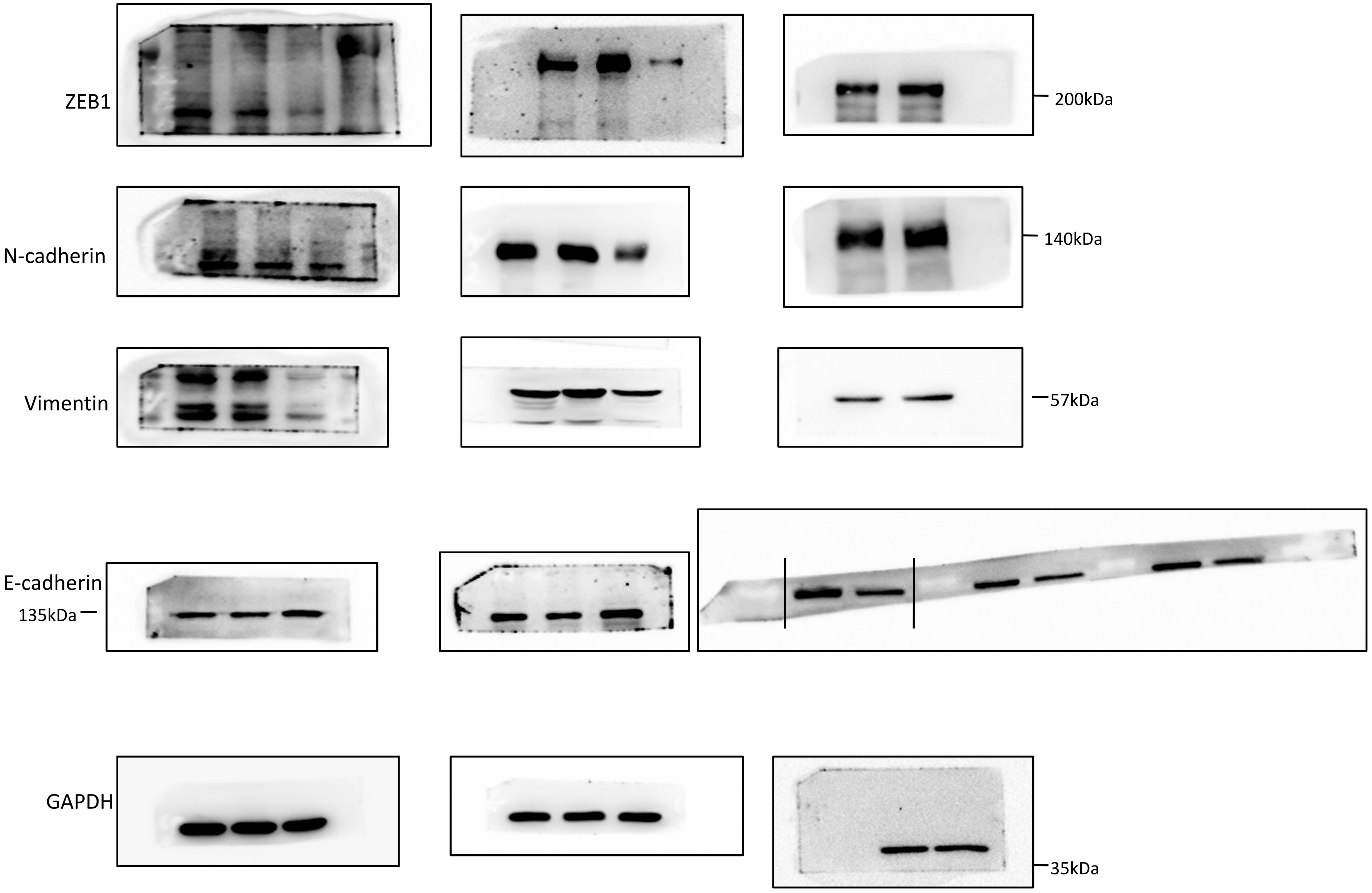

Blots for Figure 7B

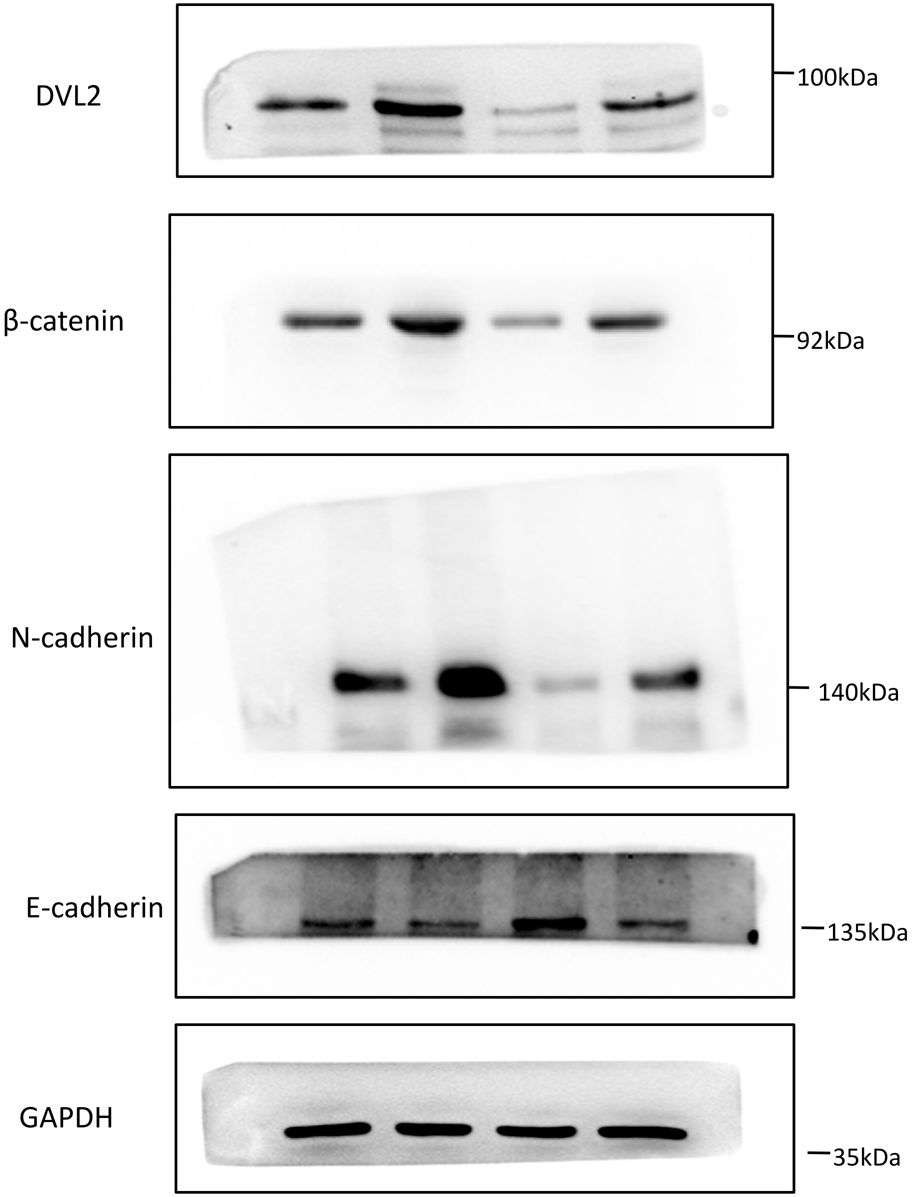

Supplement: Supplementary file 1 — Supplemental Information [file 41598_2019_44048_MOESM1_ESM.pdf]
